# The evolving landscape of epigenetic target molecules and therapies in myeloid cancers: focus on acute myeloid leukemia and myeloproliferative neoplasms

**DOI:** 10.1038/s41375-025-02639-x

**Published:** 2025-05-15

**Authors:** Michael W. M. Kühn, Naveen Pemmaraju, Florian H. Heidel

**Affiliations:** 1 Department of Hematology and Medical Oncology, University Medical Center, Johannes Gutenberg-University, Mainz, Germany; 2 German Cancer Consortium (DKTK) partner site Frankfurt/Mainz and German Cancer Research Center (DKFZ), Heidelberg, Germany; 3 Department of Leukemia, University of Texas, MD Anderson Cancer Center, Houston, TX USA; 4 Hematology, Hemostasis, Oncology and Stem Cell Transplantation, Hannover Medical School (MHH), Hannover, Germany; 5 Leibniz Institute on Aging, Fritz-Lipmann-Institute, Jena, Germany

**Keywords:** Acute myeloid leukaemia, Myeloproliferative disease, Cancer epigenetics, Molecularly targeted therapy

## Abstract

Research on myeloid neoplasms, a field that has been driving scientific advances in cancer for over 50 years, has yielded many discoveries that have fundamentally reshaped our understanding of cancer biology. These insights, often the product of leukemia research, have been instrumental in developing more mechanism-based treatments in the early 2000s [1]. Recognizing epigenetic dysregulation as a common disease mechanism in myeloid cancers has been groundbreaking regarding recent treatment developments that exploit chromatin-based oncogenic mechanisms. In the case of acute myeloid leukemia (AML), sequencing studies aimed at assessing the complement of genetic alterations demonstrated that more than 60% of AML cases harbored disease-driving mutations in epigenetic regulators. This high prevalence underscores the importance of epigenetic dysregulation in AML pathogenesis [2, 3]. Chromatin regulators commonly control disease-specific transcriptional programs, making them attractive therapeutic targets to manipulate neoplastic gene expression programs, particularly in myeloid neoplasms. Several drugs targeting epigenetic mechanisms and exploiting myeloid disease-specific dependencies have recently been approved for treating myeloid neoplasms. Many additional drugs are currently being investigated in clinical trials, and numerous new compound developments are being studied in preclinical studies. This manuscript will review (1) chromatin-based disease mechanisms, such as DNA methylation, chromatin regulatory complexes, and histone modifications, currently investigated for therapeutic exploitation in myeloid malignancies, and (2) therapeutic developments already approved or investigated for treating these diseases.

## Epigenetic targets in acute and chronic myeloid cancers

### Enzymes involved in DNA methylation (DNMT3A, TET2, IDH1, IDH2)

The first epigenetic modification identified, DNA Methylation, was initially linked to cellular differentiation processes [4]. The detection of global hypomethylation [5], and hypermethylated tumor suppressor genes in tumors and later discoveries that these and other epigenetic modifications may regulate gene expression marked the beginning of epigenetic therapy developments altering these modifications [6].

Epigenetic modifier genes such as DNMT3A (DNA methyltransferase 3 alpha) and TET2 (ten-eleven translocation 2) play pivotal roles in regulating gene expression through modification of the DNA methylation landscape. Their alterations are frequently implicated in the pathogenesis of myeloid neoplasms, including AML, myelodysplastic neoplasia (MDS), and myeloproliferative neoplasms (MPNs). Understanding the function and the impact of mutations in these genes helps elucidate the complex mechanisms underlying myeloid malignancies and provides insights into potential therapeutic targets.

DNMT3A functions as a DNA methyltransferase that adds methyl groups to cytosine residues in DNA [7]. This methylation generally leads to gene silencing. DNMT3A mutations are among the most common genetic alterations observed in AML, found in approximately 20–30% of cases [3, 8]. These mutations typically result in a loss of function, leading to DNA hypomethylation and subsequent dysregulation of gene expression. The most frequent mutation, DNMT3A R882, disrupts the enzyme’s ability to effectively methylate DNA, leading to the development and progression of myeloid malignancies by affecting the expression of genes involved in cell differentiation and proliferation [9, 10]. Recent studies provided evidence that patients with DNMT3A mutations tend to have an inferior prognosis, with increased rates of relapse and reduced overall survival compared to patients without these mutations. These mutations are often present in hematopoietic stem cells and can persist in remission, suggesting a role in disease relapse [11].

In contrast, TET2 is involved in DNA demethylation. It catalyzes the conversion of methylcytosine (5mC) to 5-hydroxymethylcytosine (5hmC), an intermediate step in the active DNA demethylation pathway [12, 13]. This modification can lead to gene activation. Mutations in TET2 are also common in myeloid cancers, particularly in MDS and MPNs, and are associated with a clonal advantage of hematopoietic cells. The loss of TET2 function results in hypermethylation and abnormal gene silencing, similar to DNMT3A mutations, albeit through a contrasting mechanism of failing to remove methyl marks (reviewed in [14]). Like DNMT3A, TET2 mutations are associated with an adverse clinical outcome in a context-dependent manner in various myeloid neoplasms [15, 16]. They can lead to an increased risk of transformation from MDS to AML and are associated with specific clinical features, such as monocytosis and splenomegaly in MPNs. The presence of TET2 mutations has also been linked to an enhanced response to hypomethylating agents in treatment settings, indicating potential therapeutic implications [17].

The interplay between DNMT3A and TET2 in myeloid neoplasms underscores a delicate balance in epigenetic regulation required for normal hematopoiesis. Mutations that disrupt this balance lead to the clonal expansion of myeloid cells and contribute significantly to the malignant phenotype. Recent research into dual mutations in *DNMT3A* and *TET2* suggests a complex interaction in which these mutations cooperate to promote leukemogenesis more effectively than either mutation alone (reviewed in [18]).

Therapeutically, targeting the epigenetic dysregulation caused by *DNMT3A* and *TET2* mutations holds promise. Current approaches include DNA methyltransferase inhibitors (also known as hypomethylating agents, HMA) and efforts to develop specific inhibitors that can restore the normal function of these epigenetic modifiers.

IDH1 and IDH2 (*isocitrate dehydrogenase 1* and *2)* are citric acid cycle enzymes that are recurrently affected by disease driving-mutations in myeloid malignancies, particularly AML (8% and 9% of cases, respectively) [2, 3, 19]. Mutated *IDH1* and *IDH2* enzymes both acquire a neo-enzyme activity leading to the accumulation of 2-hydroxyglutarate (2-HG) and a concurrent decrease in alpha-ketoglutarate (a-KG) [20, 21]. These citrate metabolizing enzymes were linked to epigenetic processes when it was discovered that 2-HG induces DNA hypermethylation and impaired differentiation in hematopoietic cells, at least partially by inhibiting the TET oncogene family demethylating enzyme function [22]. In AML, a global DNA hypermethylation pattern is similarly found in *IDH1*-mutated, *IDH2*-mutated, and *TET2*-mutated genotypes. While inducing similar DNA methylation patterns, the occurrence of *IDH* and *TET2* mutations is mutually exclusive in AML patients, further supporting a common mechanism of leukemogenesis [22]. While responses to hypomethylating agents (HMAs) in *IDH* and *TET2* mutated myeloid neoplasms did not result in much better responses than in unselected MDS/AML patients, the development of selective inhibitors of mutated *IDH1* and *IDH2* is a success story of targeted therapy in these MDS/AML genotypes. Several per oral small-molecule inhibitors have been investigated in clinical trials and induced dramatic responses in *IDH1* or *IDH2* mutated AML in various indications (reviewed in chapter 2). These inhibitors all reduce blood 2-HG levels markedly and are, therefore, likely to act via the above-proposed mechanism [23, 24].

### Epigenetic complexes and associated proteins

Epigenetic regulation involves dynamic and reversible modifications orchestrated by three classes of proteins: writers that add chemical groups (e.g., methyltransferases, acetyltransferases), readers that recognize these modifications (e.g., bromodomain proteins like ENL), and erasers that remove these marks (e.g., demethylases, deacetylases). Recent interest has expanded to include lysine acetyltransferase (KAT)-containing complexes and reader proteins such as ENL, which are integral to transcriptional elongation processes and represent additional therapeutic targets for epigenetic modulation in myeloid malignancies [6].

The KMT2A gene, also known as MLL (Mixed Lineage Leukemia) [25], plays a critical role in the pathogenesis of myeloid leukemias through its involvement in chromatin modification and gene expression regulation [26]. KMT2A encodes a histone methyltransferase primarily responsible for the methylation of histone H3 at lysine 4 (H3K4), a key epigenetic modification associated with the activation of gene transcription. This activity is crucial for properly regulating gene expression during development and hematopoiesis [27]. However, when KMT2A is disrupted or altered by chromosomal translocations [28], it leads to the formation of fusion proteins that drive the development of leukemia by hijacking normal cellular mechanisms and altering gene expression profiles [29]. KMT2A rearrangements (KMT2A-r) are prominent drivers of aggressive acute leukemias, including acute myeloid leukemia (AML), acute lymphoblastic leukemia (ALL), and leukemias exhibiting mixed-lineage phenotypes, often referred to historically as ‘mixed lineage leukemia’. KMT2A fusions are particularly prevalent in pediatric AML, where they are associated with distinct clinical and biological characteristics. These fusions result from the KMT2A gene on chromosome 11q23 being aberrantly fused with over 70 different partner genes [30]. Such translocations produce hybrid proteins that retain the N-terminal portion of KMT2A, including its AT-hooks and DNA-binding motifs. However, they replace the C-terminal SET domain, responsible for its methyltransferase activity, with various portions of the fusion partner. This structural alteration fundamentally changes the protein’s function, leading to the dysregulation of gene expression. The mechanisms through which KMT2A fusions contribute to leukemogenesis are multifaceted. First, the fusion proteins gain the ability to aberrantly recruit additional chromatin modifiers, including the super elongation complex (SEC) and the DOT1L histone methyltransferase, which are critical for the elongation phase of transcription [31]. This recruitment leads to increased transcriptional elongation at target gene loci, particularly affecting genes involved in hematopoietic regulation and development. KMT2A-rearranged leukemias characteristically exhibit marked upregulation of homeobox A (HOXA) genes, especially HOXA9, which are central to leukemic transformation. While HOXA gene upregulation is a common feature, rare KMT2A rearrangements may occur without pronounced HOXA dysregulation, highlighting a degree of heterogeneity among these leukemias. Both homeobox A (HOXA) and MEIS1 transcription factor genes are crucial for the maintenance of hematopoietic stem cells and progenitor cells, thereby contributing to leukemic transformation [32]. Second, KMT2A fusion proteins alter the chromatin landscape by changing histone modification patterns, thereby affecting the expression of a broad range of genes involved in cell cycle regulation, apoptosis, and differentiation. The loss of normal KMT2A function further exacerbates these changes, as the normal epigenetic regulation of gene expression necessary for hematopoietic differentiation is disrupted [27]. The clinical implications of KMT2A fusions in myeloid leukemia are significant. These genetic aberrations are associated with a poor prognosis, mainly due to the aggressive nature of the resultant leukemia and its resistance to conventional chemotherapies. Patients with *KMT2A*-rearranged leukemia often exhibit high leukocyte counts, widespread infiltration of organs by leukemic cells, and a higher incidence of relapse [33].

**Fig. 1:**
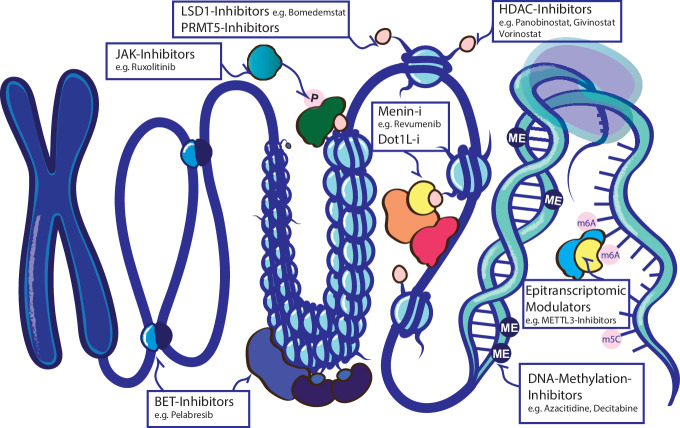
Epigenetic target structures and inhibitor classes. Current clinical development including DNA-Methylation Inhibitors, Menin-/Dot1L-Inhibitors, BET-Inhibitors, HDAC-Inhibitors, LSD1/PRMT5-Inhibitors and indirect effects of JAK-Inhibitor treatment.

**Menin**, a protein encoded by the *MEN1* (multiple endocrine neoplasia 1) gene, is critically involved in various cellular processes, including transcriptional regulation, proliferation, and differentiation. It is known primarily for its role in the similarly named multiple endocrine neoplasia type 1 syndrome, a familial syndrome with autosomal dominant inheritance, where affected patients develop tumors in various endocrine glands. These patients carry germline mutations in the *MEN1* gene, and therefore, Menin is considered a tumor suppressor gene in endocrine tissues [34]. However, several studies have demonstrated that Menin serves as an oncogenic co-factor in specific subtypes of acute leukemia, underpinning the highly context-specific function of this protein. Menin itself has no enzymatic function and does not harbor a chromatin-binding domain, but it is a nuclear protein that functions as an adaptor within the KMT2A chromatin complex [35]. The association of oncogenic KMT2A-fusion proteins with Menin and LEDGF is required for its chromatin binding, target gene expression such as the *HOXA9* and *MEIS1*, and implicated the Menin-KMT2A interaction as a therapeutic opportunity in these leukemias [36, 37]. Lens Epithelium-Derived Growth Factor (LEDGF), also termed PSIP1, is a transcriptional co-activator crucial for tethering proteins such as KMT2A fusion proteins to chromatin, thereby facilitating aberrant transcriptional activation of leukemic target genes such as HOXA9 and MEIS1. Jolanta Grembecka’s group was the first to synthesize small molecules targeting the KMT2A binding site in Menin to specifically inhibit this protein-protein interaction (Fig. 1) and demonstrated activity in *KMT2A*-rearranged leukemia models [38]. We recently demonstrated that the aberrant expression of *MEIS1, PBX3*, and several *HOX* transcription factor genes in the *NPM1* mutated AML depend on the Menin-KMT2A (wildtype) protein interaction, represent a therapeutic opportunity and early Menin inhibitors had activity against preclinical models of this leukemia subtype [39]. Shortly after those reports, several companies developed clinical-grade oral Menin inhibitors with higher specificity and potency that had dramatic effects in murine models of *KMT2A*-rearranged and *NPM1*-mutated AML and eradicated disease in PDX models [40–43]. All of these menin inhibitors that effectively disrupt the menin-MLL interaction lead to decreased expression of KMT2A target genes, inducing differentiation and apoptosis of leukemic cells, and have dramatic activity in early clinical trials in patients with heavily pretreated relapsed or refractory *KMT2A*-rearranged or *NPM1*-mutated AML [44, 45].

Polycomb repressive complexes (PRC), particularly PRC1.1, act antagonistically to the KMT2A complex by catalyzing chromatin compaction and transcriptional repression, thereby counterbalancing KMT2A-mediated gene activation and contributing to the intricate regulatory balance disrupted in leukemogenesis. The Polycomb-repressive complex 2 (PRC2) catalytic subunit *enhancer of zeste 2* (EZH2) is a H3K27-specific methyltransferase to maintain the formation of H3K27me3, a mark associated with transcriptional repression. The PRC2 complex can be seen as a functional counterpart to the KMT2A complex and has, for instance, been demonstrated to be essential for the regulation of HOX transcription factor expression during development. EZH2 is commonly affected (activating) mutations in various tumors and the specific small-molecule inhibitor Tazemetostat has been approved for the treatment of follicular lymphoma. One preclinical study implicated EZH2 in the pathogenesis of a murine *KMT2A-MLLT3* AML model, but generally, EZH2 does not seem to represent an important driver in the pathogenesis of myeloid neoplasms and is currently not considered a promising therapeutic target these diseases [46, 47].

### Other Histone in these diseases

**LSD1** (lysine-specific demethylase 1), also known as KDM1A, is an enzyme involved in the regulation of gene expression through epigenetic modification. Its primary molecular function includes its ability to demethylate lysine residues on histone proteins, which impacts the transcriptional activity of genes [48, 49]. LSD1 specifically demethylates mono- and di-methylated lysine 4 (K4) and lysine 9 (K9) on histone H3. The demethylation of these residues can lead to either transcriptional repression or activation, depending on the site of demethylation and the broader chromatin context. When LSD1 demethylates mono- and di-methylated H3K4 (H3K4me1 and H3K4me2), it is generally believed to result in transcriptional repression. H3K4 methylation is typically associated with active transcription and its inactivation can silence gene expression. Moreover, LSD1 can demethylate H3K9me1/2 in a context-dependent manner, which is generally associated with transcriptional activation, as H3K9 methylation is a marker of gene repression. LSD1 belongs to the flavin adenine dinucleotide (FAD)-dependent amine oxidase family. It catalyzes the oxidative demethylation of methylated lysine residues using FAD as a cofactor. This process involves the oxidation of the methyl group, leading to its release as formaldehyde. The activity of LSD1 is modulated by its interaction with various co-repressors and co-activators [49]. For example, it forms a complex with CoREST (co-repressor for element-1-silencing transcription factor), which enhances its ability to repress transcription. The Nucleosome Remodeling and Deacetylase (NuRD) complex influences myeloid cancers by regulating gene expression and maintaining genomic stability. Loss of MBD3, a NuRD subunit, has been linked to leukemogenesis, suggesting a tumor suppressor role in myeloid leukemia [48]. Additionally, depletion of CHD4, another NuRD component, sensitizes AML cells to DNA-damaging agents, indicating its involvement in DNA repair mechanisms within these malignancies. By altering the methylation status of histones, LSD1 can affect chromatin structure and thus regulate access of the transcriptional machinery to DNA. This is a key aspect of its role in controlling gene expression. Beyond histones, LSD1 is known to demethylate non-histone proteins, influencing their function and stability. This broadens its impact on cellular processes. In hematologic cancers, aberrant LSD1 activity can disrupt the normal patterns of gene expression that are crucial for blood cell development and function. LSD1 is involved in the regulation of hematopoiesis, the process by which blood cells are formed. Its dysregulation can contribute to the development of hematologic malignancies by promoting the proliferation and survival of malignant cells and inhibiting their differentiation [50]. LSD1 has been particularly implicated in subtypes of leukemia, such as AML [51]. LSD1 can be overexpressed or functionally altered, contributing to leukemogenesis by affecting the expression of genes involved in cell cycle regulation, apoptosis, and differentiation [52]. Given its critical role in the pathogenesis of hematologic cancers, LSD1 has emerged as a potential therapeutic target. Inhibitors of LSD1 are being explored as treatments, with the idea that inhibiting LSD1 could restore normal patterns of gene expression, induce differentiation, and inhibit the growth of cancerous cells. LSD1 may interact with other oncogenes or tumor suppressors to drive cancer progression. In some cases, the function of LSD1 is altered by its interaction with fusion proteins or other oncogenic factors common in hematologic cancers [53, 54].

**DOT1L** (Disruptor of Telomeric silencing 1-Like) is associated with KMT2A fusion proteins (Fig. 1) in KMT2A-rearranged AML, as it interacts with many of the known fusion partner proteins of KMT2A, such as AFF1 (also known as AF4), and MLLT3 (also known as AF9). As the only known histone methyltransferase that catalyzes the methylation of lysine 79 of histone H3 (H3K79), leading to the formation of H3K79 di- and trimethylation (me2/me3), DOT1L is integral to the regulation of gene expression through epigenetic mechanisms [55]. The methylation of H3K79 influences chromatin structure and, consequently, the transcriptional activity of genes critical for cell proliferation and differentiation. In the context of AML with KMT2A rearrangements, DOT1L’s enzymatic activity becomes pathophysiologically significant. As described earlier, KMT2A-fusion proteins aberrantly recruit chromatin modifiers such as DOT1L, resulting in sustained HOXA9 and MEIS1 expression, which drives leukemogenesis by promoting proliferation and blocking differentiation [56]. In normal cells, the expression of these genes is tightly regulated and restricted to specific stages of cell development [57]. However, in leukemic cells, sustained expression of these genes due to aberrant DOT1L activity contributes to the blockage of cellular differentiation and the promotion of unchecked cellular proliferation, hallmark features of leukemia. The critical role of DOT1L in driving oncogenic gene expression programs has made it a target for therapeutic intervention. Small-molecule inhibitors of DOT1L have been developed and are currently under clinical investigation [56]. These inhibitors specifically target the methyltransferase activity of DOT1L [58], aiming to reverse the aberrant methylation and associated transcriptional programs that sustain leukemic cell growth and survival.

**PRMT5** (protein arginine methyltransferase 5) is an epigenetic modifier belonging to the family of protein arginine methyltransferases (PRMTs, currently known as PRMT1-11) that set post-transcriptional modifications at histone and non-histone proteins. While most histone-modifying enzymes are lysine-specific, PRMTs catalyze methylation on arginine residues. PRMT5 belongs to the type II PRMTs that generate two of three types of methylated arginine residues, monomethyl-arginine (MMA) and symmetric dimethylarginine (sDMA) [59]. PRMT5 forms a larger protein complex with either WDR77 or MEP50, and its association with one of these proteins is required for active enzyme function [60]. Although PRMT5 modifies also non-histone proteins, several reports indicate that it may be a binding partner or associated with the nuclear protein Menin. PRMT5 has been demonstrated to alter H2AR3, H3R2, H3R8, H3R2, and H4R3, while it may have a preference for histone 4. The symmetrical methylation of these residues is associated with transcriptional regulation, in most cases with gene silencing [61–63].

Besides transcriptional regulation via histone modification, PRMT5 has been implicated in multiple cellular processes, including alternative splicing, mRNA translation, and the DNA damage response [64]. It has also been linked to the pathogenesis of different cancer types, including solid tumors, lymphomas, and myeloid neoplasms [65, 66]. Various inhibitors that block the PRMT5 enzyme function have been developed and are currently under clinical investigation for the treatment of many different tumors, including myeloid malignancies.

In AML, PRMT5 contributes to leukemogenesis by silencing key regulatory genes. Notably, PRMT5 suppresses the expression of SP1 and FLT3 through the repression of miR-29b [67]. Moreover, PRMT5 plays a pivotal role in safeguarding alternative splicing by regulating serine/arginine-rich splicing factor 1 (SRSF1), a crucial component of the splicing machinery [68]. Additionally, PRMT5 interacts with JAK2, leading to the downregulation of E2F target genes, further promoting leukemogenesis [69].

In chronic myeloid leukemia (CML), PRMT5 is consistently overexpressed, contributing to disease progression and resistance to therapy [70]. Furthermore, recent studies highlight a novel connection between PRMT5 and polo-like kinase 4 (PLK4) in AML with TP53 mutations, wherein PRMT5 phosphorylation modulates leukemic cell survival and proliferation [71]. Given its diverse regulatory functions, PRMT5 emerges as a promising therapeutic target in hematologic malignancies. Ongoing research into PRMT5 inhibitors may provide novel strategies to counteract its oncogenic effects, offering new avenues for targeted therapies in AML, CML, and other myeloid neoplasms.

### BET-proteins

BET proteins, particularly bromodomain-containing protein 4 (BRD4), are critical regulators of gene expression and chromatin dynamics [48]. Through their tandem bromodomains, BET proteins recognize acetylated lysines on histone tails, facilitating the recruitment of transcriptional machinery and maintenance of transcriptional elongation by RNA polymerase II. BRD4 uniquely remains associated with chromatin during mitosis, enabling transcriptional memory in cell division. This epigenetic function plays a pivotal role in regulating key oncogenic pathways, including MYC and NF-κB, and maintaining cellular homeostasis [6].

In hematologic neoplasms, aberrant BRD4 activity contributes to dysregulated transcriptional programs that drive malignancy. In AML, BRD4 supports leukemogenesis by stabilizing MYC expression and activating anti-apoptotic pathways. Similarly, in myeloproliferative neoplasms (MPNs) and lymphoma, BRD4 is implicated in the activation of inflammatory cytokine signaling and abnormal cell proliferation [72].

Pharmacologic targeting of BET proteins, particularly with BET inhibitors (BETis), has shown therapeutic promise [48]. Compounds such as JQ1 and CPI-0610 disrupt BET protein interactions with acetylated histones, downregulating oncogenic transcriptional networks. In clinical trials, BETis have effectively reduced tumor burden and modulated immune microenvironments in hematologic malignancies.

### Epitranscriptomic modifiers, including RNA methylation

Epitranscriptomic modifiers are enzymes and proteins that modify and/or bind to RNA molecules post-transcriptionally, without altering the RNA sequence itself [73, 74]. This emerging field has evolved in addition to modifications of DNA and histones that affect gene expression. Modifications introduced by epitranscriptomic modifiers regulate various aspects of RNA metabolism and function. Over 100 different types of chemical modifications are known to occur on RNA molecules. Among the most studied modifications are N6-methyladenosine (m6A), 5-methylcytosine (m5C), pseudouridine (Ψ), and inosine (I). Each of these modifications can influence RNA stability and function in distinct ways [73, 74]. First, epitranscriptomic modifications can regulate gene expression through RNA splicing, export from the nucleus, RNA stability, and translation efficiency. As one example, m6A modification on mRNA can promote or inhibit translation depending on the context and the proteins that recognize this modification. Epitranscriptomic RNA modifications such as m6A can affect the splicing of pre-mRNA and the stability of mature mRNA, leading to changes in the types and quantities of proteins produced in the cell [75]. Dynamic changes in these modifications are also critical during developmental processes, for cell fate decisions and differentiation programs at the stem- and progenitor cell level. Likewise, cells can use RNA modifications to respond to cellular stress, rapidly. Here, by altering the pattern of RNA modifications, cells quickly adjust the expression of stress response genes. Dysregulation of epitranscriptomic modifications has been implicated in a range of diseases, including malignant transformation, (hematologic) cancers, and degenerative diseases (such as neurological disorders or metabolic diseases). During malignant transformation, abnormal RNA modification patterns can lead to the dysregulation of oncogenes and tumor suppressor genes.

Currently, targeting epitranscriptomic modifiers is being explored as a therapeutic strategy in various diseases. METTL3 is the core catalytic component of the m6A methyltransferase complex (Fig. 1). It catalyzes the transfer of a methyl group to the N6 position of adenosine residues in RNA. Through its role in m6A RNA methylation, METTL3 indirectly influences gene expression. METTL3 inhibitors work by binding to the METTL3 enzyme and preventing it from catalyzing the m6A modification on RNA. This inhibition results in changes in the expression and function of genes that are regulated by m6A.

YBX1, which acts as an m5C reader, interacts with IGF2BPs to stabilize key oncogenic transcripts such as MYC and BCL2 in AML [76, 77]. Its m6A-dependent regulation of RNA stability enhances cell proliferation and survival. In myeloproliferative neoplasms (MPN) [78], YBX1 contributes to disease persistence, making it a therapeutic target in both AML and MPN. The inhibition of FTO, ALKBH5, and YTH family proteins (YTHDF1/2 and YTHDC1) holds therapeutic promise in hematologic malignancies. FTO and ALKBH5, as m6A demethylases, modulate oncogenic mRNA stability and translation, while YTH readers regulate mRNA fate through binding m6A-modified transcripts. Targeting FTO enhances leukemic cell apoptosis, inhibits proliferation, and sensitizes cells to chemotherapy. ALKBH5 inhibition disrupts stemness and tumor progression by destabilizing m6A-modified oncogenic transcripts. Inhibiting YTHDF1/2 or YTHDC1 blocks m6A-mediated transcript stabilization, impacting pathways critical for leukemia stem cell survival, and highlighting their potential in epigenetic therapies for aggressive hematologic cancers [79].

### Signaling into chromatin

Aberrant JAK2 signaling exerts profound effects on the epigenetic landscape through direct histone modifications and secondary mechanisms [80, 81]. Nuclear JAK2 phosphorylates histone H3 at tyrosine 41 (H3Y41), displacing HP1α (CBX5) from chromatin, which promotes euchromatin formation at promoters, gene bodies, and cis-regulatory elements [82, 83]. This pathway regulates the expression of critical genes such as NFE2 [84], overexpressed in most myeloproliferative neoplasms (MPNs), and NANOG [85], essential for cytokine independence. Additionally, JAK2 phosphorylates the histone demethylase KDM3A, enabling the removal of H3K9 methylation at STAT3 binding sites, and ensuring a permissive chromatin environment [86]. Histone demethylases KDM4C and KDM3C were recently identified as a signaling effector of mutated JAK2 and a target gene of the transcription factor NFE2, which is overexpressed in myeloproliferative neoplasms [84, 87, 88].

Histone acetylation represents another key layer of JAK2-driven epigenetic dysregulation (Fig. 1). Activated STAT proteins recruit acetyltransferases, including p300, CBP [89–91], and TIP60 [92], to chromatin, enhancing transcriptional activity at inflammatory pathway-regulated loci such as TNFα-NFκB [93–95]. This aberrant acetylation recruits bromodomain proteins like BRD4, which sustain inflammatory gene expression programs and MPN cell fitness. Pharmacologic inhibition of BET proteins or HDACs [96] effectively silences this aberrant signaling, with particular sensitivity observed in CALR-mutant MPNs. Beyond chromatin, JAK2-V617F impacts DNA methylation by phosphorylating TET2, leading to global hypomethylation [97].

These findings highlight JAK2’s multifaceted role of in driving epigenetic dysregulation in MPNs and underscore its therapeutic potential for targeted interventions.

## Targeting the epigenetic machinery in acute myeloid leukemia

### Hypomethylating Agents (Azacitidine, Decitabine, CC-486, ASTX727)

The DNA hypomethylating agents (HMAs) **decitabine (DEC)** and **azacitidine (AZA)**, which inhibit DNA methyltransferases (Fig. 1), were the first non-selective epigenetic drugs approved for the treatment of AML and MDS with an excess of blasts.

Their use as single agents was long considered a standard-of-care treatment for AML patients not eligible for intensive chemotherapy. Randomized trials assessing decitabine monotherapy (5 days) versus mostly low-dose Ara-C (LDAC) resulted in higher CR rates (17.8% vs. 7.8%) and a non-significant trend towards improved overall survival (OS, median: 7.7. vs. 5.0 months) [98]. AZA single-drug treatment resulted in an OS increase over three conventional care regimens (mainly consisting of LDAC) in the AZA-AML-001 trial (10.4 vs. 6.5 months, *p* = 0.1) [99]. Although HMA treatment is generally not considered a curative treatment option for AML patients who are fit for intensive treatment, a recent randomized trial compared an intensified 10-day DEC regimen with conventional 7 + 3 intensive chemotherapy in elderly fit unselected AML patients ≥ 60 years who were intended to receive consolidation therapy with allogenic stem cell transplantation. In the overall cohort, DEC was not significantly inferior to the conventional 7 + 3 arm (4-year OS: 26% vs. 30%, p = 0.68) with a median survival of only 15 vs. 18 months, respectively [100].

While HMA treatment alone still has very limited activity against AML, AZA has become indispensable as a combination partner for the BCL2-inhibitor venetoclax (VEN) or the IDH1 inhibitor ivosidenib (IVO, see below). In randomized phase-III trials on previously untreated elderly non-fit AML patients these regimens significantly enhanced composite CR rates (AZA/VEN: 66.4% vs. AZA: 28.3; AZA/IVO: 54% vs. AZA: 16%) and median OS (AZA/VEN: 14.7 vs. AZA: 9.6; AZA/IVO: 29.3 vs. AZA: 7.9 months) in unselected AML and IDH1 mutated AML patients, respectively [101, 102]. While these AZA-containing doublet regimens are considered the current standard of care regimens for non-intensive treatment, the current clinical investigation focuses on the assessment of adding a third targeted drug to these AZA-based doublet regimens for enhancing survival rates of non-intensive AML treatment.

More recently, novel drug formulations of the parenterally administered HMAs were developed and approved in specific indications for AML treatment based on randomized trials. An oral **decitabine/cedazuridine** formulation [103, 104] has now been approved for monotherapy treatment of high-risk MDS (U.S.) and AML monotherapy (Europe) (Table 1). Also, an orally administered version of **azacitidine (CC-486)** has been approved as maintenance after intensive chemotherapy. This formulation reduced relapse risk and prolonged survival (median OS from 14.8 to 24.7 months) of elderly patients not considered candidates for allogeneic SCT. Future studies will determine if these oral formulations can replace conventional DEC or AZA in combination regimens.

**Table 1:** Examples of epigenetic inhibitors in current clinical development.

Inhibitor Target /Class	Drug Name	Alternative Drug Names	Clinical Trial Phase (ongoing / completed)*	Myeloid Neoplasm Trial Indication [Trial Phase]	Approval [Y/N, Year (FDA/EMA)]	Approval Indication**
BET-Inhibitors	Pelabresib	CPI-0610	1–3 (completed)	Myeloid neoplasms [1/2], MF [1–3]	N	N.A.
	Ezobresib	BMS-986158	1/2 (completed)	MF	N	N. A.
DOT1L-Inhibitor	Pinometostat	EPZ-5676	1/2, 2 (completed)	AML (*KMT2A*-r)	N	N. A.
HDAC-Inhibitors	Givinostat	ITF-2357	2–3 (ongoing)	PV [2,3]	N	N. A.
	Panobinostat	LBH589	2 (completed)*	CML, MDS, MF	N	N. A.
	Vorinostat	Suberoylanilide Hydroxamic Acid	1/2, 2 (completed)	AML [2], MDS [1/2]	N	N. A.
Hypomethylating Agents (HMAs)	Azacitidine	5-Azacytidine	1, 1/2 (ongoing) 3 (completed)	AML, MDS, MDS/MPN	Y (2004/2008)	MDS, JMML (FDA); MDS, AML (EMA)
	Oral Azacitidine	CC-486	1–2 (ongoing), 3 (completed)	AML, MDS, MPN	Y (2020/2021)	AML (maintenance)
	Decitabine	5-Aza-2′-deoxycytidine	1–3 (completed), 1/2 (ongoing) 3 (not yet recruiting)	AML, MDS, MPN	Y (2006/2012)	MDS (FDA) AML (EMA)
	Oral decitabine/ cedazuridine	ASTX727	1–3 (completed) 1/2 (ongoing)	AML, MDS	Y (2020/2021)	MDS (FDA) AML (EMA)
IDH-Inhibitors	Enasidenib (IDH2)	AG-221	1–3 (completed)1-2 (ongoing)	AML (*IDH2*-mutated) [1–3] MDS (*IDH2*-mutated)	Y (2017)	*IDH2*-mutated R/R AML (FDA)
	Ivosidenib (IDH1)	AG-120	1–3 (completed) 1–3 (ongoing)	AML (*IDH1*-mutated) MDS (*IDH1*-mutated)	Y (2018 and 2022/2023)	*IDH1*-mutated R/R AML (FDA); *IDH1*- AML (FDA/EMA)
	Olutasidenib (IDH1)	FT-2102	1/2 (completed) 1–2 (ongoing)	AML (*IDH1*-mutated), Myeloid Neoplasm (*IDH1*-mutated)	N (2022)	*IDH1*-mutated R/R AML (FDA)
LSD1-Inhibitors	Bomedemstat	MK-3543, IMG-7289	1/2 (completed) 1–3 (ongoing)	Myeloid Neoplasms, ET, MF, PV [1/2], ET [3]	N	N. A.
Menin-Inhibitors	Bleximenib	JNJ-7527661	1–3 (ongoing)	AL (*KMT2A*-r, *NPM1*-mutated, *NUP98*-r) [1–3] AML, *HOX*-high AML [1,2]	N	N. A.
	Enzomenib	DSP-5336	1/2 (ongoing)	AL (*KMT2A*-r, *NPM1*-mutated)	N	N. A.
	Revumenib	SNDX-5613 (close [preclinical] homolog: VTP-50469)	1/2 (completed) 1–3 (ongoing)	AML (*KMT2A*-r, *NPM1*-mutated) [1–3] AML, *HOX*-high AML [1,2]	Y (2024)	R/R *KMT2A*-r AL (FDA)
	Ziftomenib	KO-539	1 (completed) 1–2 (ongoing)	AML (*KMT2A*-r, *NPM1*-mutated, *NUP98*-r) [1–3] AML, *HOX*-high AML [1,2]	N	N. A.
PRMT5-Inhibitors	Pemrametostat	GSK3326595, EPZ015938	1/2 (completed)	AML, MDS	N	N. A.
	Onametostat	JNJ-64619178	1/2 (completed)	MDS	N	N. A.

*Only referring to trials assessing myeloid neoplasms, in which at least exploratory efficacy data have been reported.

**newly diagnosed unless specifically indicated otherwise; *R/R* relapsed refractory.

# One phase 1/2 trial (NCT01298934) with “unknown” trial status.

*KMT2A-r* KMT2A-rearranged, *AL* Acute Leukemia.

### Inhibitors of Mutated Isocitrate Dehydrogenase 1 and 2 (IDH1 and IDH2)

The first selective oral inhibitors of mutant IDH1 and IDH2 were **ivosidenib (IVO)** and **enasidenib**. Both drugs were approved for the single-drug treatment of relapsed or refractory IDH1 or IDH2 mutated AML patients, based on non-randomized phase II trials [23, 24] in the U.S. and resulted in combined CR rates of 30.2% and 26.1% as oral single agents, respectively (Table 1). Very recently, **olutasidenib**, a novel potent and selective oral inhibitor of IDH1, was reported to induce combined CR rates of 35% in heavily pretreated IDH1 mutated AML patients in a phase-II trial and subsequently approved for treatment in the U.S. Ivosidenib and enasidenib were also quickly introduced into first-line treatment of AML patients and both drugs induced durable remissions in combination with AZA in previously untreated elderly patients, not fit for intensive treatment: As indicated above, in a randomized phase III trial on IDH1 mutated AML patients, the combination of IVO and AZA increased combined CR rates from 16% (AZA alone) to 54% and median OS from 7.9 to 29.3 months [102].

Based on these data, the combination of IVO/AZA was approved in the U.S. and Europe for this indication. However, a randomized phase-II trial assessing the combination of enasidenib and AZA vs. AZA alone did not reach its primary OS endpoint.

While IDH2 mutated AML patients responded significantly better with an overall response rate of 74% vs 36% (*p* = 0.0003), overall survival was similar between the treatment groups, likely influenced by patients progressing on AZA receiving the already approved enasidenib as a second-line treatment [105].

Early clinical trials of IDH1/IDH2 inhibitors with induction chemotherapy have shown promising results, instilling a sense of optimism to increase responses and survival of IDH1-mutated AML patients fit for intensive chemotherapy [106]. Results from phase-III studies (e.g., NCT03839771) designed to validate these findings in a randomized, double-blinded manner are expected soon.

### Menin Inhibitors

The dramatic activity of Menin inhibitors in preclinical models quickly led to clinical investigation of these drugs in *KMT2A*-rearranged and *NPM1*-mutated AML. Currently, five different Menin inhibitors are being investigated as single agents or in combinations in various indications in clinical trials.

A first Phase I trial (AUGMENT-101) assessed the oral single-agent revumenib (SNDX-5613, a close homolog to VTP50469) in heavily pretreated patients (*n* = 68) with relapsed or refractory *KMT2A*-rearranged or *NPM1*-mutated AML [44]. This first-in-human dose escalation trial raised attention within the scientific community as exploratory efficacy analyses demonstrated significant anti-leukemic activity in heavily pretreated patients. The median number of prior therapies was four, and 46% of the patients had relapsed following allogeneic stem cell transplantation. Revumenib was well tolerated, with only 16.2% of patients experiencing any grade 3 or higher treatment-related adverse event, the most common being QT prolongation (13%).

Of 60 patients available for the efficacy analysis, the overall response rate (ORR, excluding partial responses) was 53%, with a 30% rate of complete remissions or complete remissions with partial hematologic recovery (CR/CRh). 78% of the CR/CRh patients achieved minimal residual (MRD) negativity, and the median duration of any response was 9.1 months. Based on the exploratory efficacy assessment from the phase I, the drug received breakthrough designation from the FDA, and the AUGMENT-101 trial subsequently entered phase II. Issa and colleagues recently reported the safety and efficacy evaluation for the *KMT2A*-rearranged AML cohort [45]. Grade≥3 adverse events in n = 94 evaluable patients included febrile neutropenia (37.2%) and QT prolongation (13.8%), while grade≥3 differentiation syndrome, a now-known class effect of Menin inhibitors, was observed in 16% of patients. In *n* = 57 efficacy-evaluable patients, the ORR was 68.2%, the CR/CRh rate was 22.8%, with 70% of patients achieving MRD-negativity. Following a priority review of these data, the FDA approved revumenib as a treatment for *KMT2A*-rearranged acute leukemias in the relapsed or refractory setting while awaiting the final analysis for the *NPM1*-mutated AML subset (Table 1).

Promising exploratory efficacy data were also published from another phase-Ia/Ib trial (KOMET-001) assessing the Menin inhibitor ziftomenib in heavily pretreated relapsed or refractory AML, which also led to the assignment of breakthrough designation status from the FDA [107]. Of 83 evaluable patients, the most common grade≥3 treatment-emergent adverse events were anemia (24%), febrile neutropenia (22%), pneumonia (19%), and differentiation syndrome (15%), but no QT prolongation. Of 36 patients with *KMT2A*-rearranged or *NPM1*-mutated AML that were treated with the recommended phase-2 dose, the CR/CRh-rate was 25%, and 35% in the *NPM1*-mutated AML cohort, respectively [107]. Phase 2 trial assessment of ziftomenib in these leukemia subtypes is ongoing.

Additional three other Menin inhibitors are currently being investigated in early trials (bleximenib, also known as JNJ-75276617 [e.g. in NCT04811560], enzomenib, also known as DSP-5336 [e.g. in NCT04988555], and BMF-219 [in NCT05153330]). Promising exploratory efficacy in relapsed or refractory *KMT2A*-r and *NPM1*-mutated AML was recently reported from ongoing analyses for the oral single-agents bleximenib and enzomenib with CR/CRh rates of 48%, 63%, with both showing a beneficial safety profile. Full data sets for publication are still pending [108, 109].

Given the strong anti-leukemic activity of these compounds, Menin inhibitors, including BN-104 (Revumenib), have demonstrated remarkable clinical efficacy in heavily pretreated relapsed or refractory (r/r) AML patients harboring KMT2A rearrangements or NPM1 mutations. Current clinical evaluations focus primarily on r/r settings, with investigations into first-line applications ongoing. Astonishing response rates have been reported for ziftomenib, bleximenib, and revumenib in combination with 7 + 3 chemotherapy or azacitidine/venetoclax (AZA/VEN), respectively.

Examples are early clinical trials assessing 7 + 3 chemotherapy in combination with ziftomenib or bleximenib as first-line treatment, resulting in CR/CRh rates of 91% and 81%, in younger patients with *KMT2A*-rearranged or *NPM1*-mutated AML [109, 110]. Similarly, in elderly unfit, previously untreated *KMT2A*-rearranged or *NPM1*-mutated AML patients, the BEAT-AML consortium reported data from an ongoing phase-I trial demonstrating an ORR (excluding PR) of 100% and a CR/CRh rate of 77% for the combination of revumenib with AZA/VEN [111]. Based on these astonishing data, a pan-European/worldwide academic consortium (EVOLVE investigators) is currently initiating a randomized, double-blinded, phase-III study, assessing the backbone of VEN/AZA with and without revumenib.

### Anti-leukemic efficacy of drugs targeting other epigenetic regulators (DOT1L, PRMT5, LSD1, BET, HDAC)

As DOT1L emerged as an essential molecular drug target in acute leukemias harboring a KMT2A-fusion protein, small-molecule inhibitors targeting DOT1L were developed to reverse aberrantly expressed leukemic transcriptional programs in those leukemias. **Pinometostat**, a highly specific and potent competitive inhibitor of the DOT1L H3K79 methyltransferase, was efficient in preclinical models but had poor oral bioavailability and a short half-life in vivo. In a Phase I single-agent trial on heavily pre-treated patients with relapsed or refractory *KMT2A*-rearranged acute leukemia, pinometostat was therefore administered as a continuous intravenous infusion in 28-day cycles. While the drug was very well tolerated and dose-limiting toxicity was not reached in any of the six dose cohorts, exploratory efficacy was modest, with only two of 51 patients achieving complete remission [58]. Given the challenges with administering pinometostat and its limited activity in a rare leukemia subtype, clinical development of pinometostat as a single agent was stopped but introduced into trials assessing its combination with AZA or induction chemotherapy (NCT03701295, NCT03724084). An exploratory efficacy analysis of those studies is pending.

Based on the preclinical data described above, a number of pharmaceutical companies developed small-molecule inhibitors to inhibit the arginine methyltransferase activity of PRMT5. While various PRMT5 inhibitors are currently under clinical investigation in various oncologic indications, two drugs were also assessed in early clinical trials on patients with myeloid malignancies. **GSK3326595** was evaluated in phase I/II trials on 30 patients with relapsed or refractory MDS, CMML, or AML. The activity was limited in this heavily pre-treated heterogeneous patient cohort, with only one patient exhibiting a complete marrow response and four patients with stable disease [112]. In a phase I dose escalation trial, another PRMT5 inhibitor, **JNJ-64619178**, was assessed on 24 patients with lower-risk MDS. With a median treatment duration of 3.45 months, no dose-limiting toxicity was observed, and no objective hematologic improvement or objective response was observed in this low-risk patient population [113] (Table 1).

Early clinical trials assessing other drugs targeting the epigenetic mechanisms indicated above, specifically non-selective histone deacetylases (panobinostat, vorinostat, pracinostat) [114–116] and one inhibitor of LSD1 (GSK2879552), showed minimal efficacy in early clinical trials assessing relapsed or refractory AML [117]. Data assessing drugs targeting these mechanisms may have a more promising role in the treatment of MPNs, and therefore, they are discussed in the MPN section of this paper.

However, the limited efficacy of some epigenetic drugs observed in early clinical trials may reflect the fact that those drugs commonly require several cycles of treatment until treatment responses are seen. As these novel drugs are widely tested in advanced-stage relapsed or refractory AML patients in high need of quick responses, delayed clinical benefits may remain unnoticed in such patient cohorts. Also, drugs aimed at targeting distinct epigenetic mechanisms are – other than chemotherapy or non-selective epigenetic drugs such as HMAs - unlikely to be active in unselected AML patient cohorts. Eliminating the possibility of false clinical failure of certain novel epigenetic therapies due to those reasons will require a more detailed preclinical characterization of drug responses and modifications to future early clinical trial testing. Menin inhibitor development and clinical investigation should be viewed as a role model for such clinical assessments.

## Epigenetic modulation in chronic myeloid neoplasms: Focus on MPN

Epigenetic dysregulation is a hallmark of chronic myeloid cancers such as myeloproliferative neoplasms (MPNs) [80, 118], driving disease progression and resistance to conventional therapies [118]. Epigenetic inhibitors, targeting key mechanisms such as DNA methylation, histone acetylation, and bromodomain function, offer novel therapeutic avenues. Agents like BET inhibitors and HDAC inhibitors have shown promise by modulating aberrant gene expression, reducing inflammation, and altering the bone marrow microenvironment. In MPNs, these inhibitors synergize with JAK inhibitors to enhance clinical outcomes by targeting both malignant clones and inflammatory signaling. Ongoing trials aim to validate their efficacy, marking a transformative shift in the treatment landscape of chronic myeloid neoplasms. Of note, therapeutic strategies that not only alleviate symptoms but also alter the disease’s natural progression are clearly needed. In this context, epigenetic drugs show promise in modifying disease pathways and improving clinical outcomes for myelofibrosis patients [119].

### Bromodomain/BET-Inhibitors

Recent studies explored the impact of dual targeting oncogenic JAK/STAT signaling and inflammatory NF-κB pathways in myeloproliferative neoplasms (MPNs) [120]. Utilizing preclinical models, the research identifies chromatin landscape alterations driven by constitutive JAK2 activation, which enhance NF-κB signaling and chronic inflammation, a hallmark of MPN pathogenesis. BET bromodomain inhibition via JQ1 effectively attenuated NF-κB-induced cytokine production and reduced bone marrow fibrosis. When combined with JAK inhibitors, BET inhibition yielded synergistic effects, significantly decreasing inflammatory cytokines, reversing fibrosis, and improving disease outcomes. This dual-targeted approach not only mitigated MPN-associated inflammation but also addresses limitations of JAK inhibitors, including persistence and minimal fibrosis impact. These findings provided a compelling rationale for clinical trials exploring combined JAK/BET inhibition to achieve transformative therapeutic outcomes for MPN patients.

***Pelabresib (CPI-0610)****,* an oral bromodomain and extra-terminal domain (BET) inhibitor, represents a promising therapeutic advancement for myelofibrosis (MF), a myeloproliferative neoplasm driven by JAK-STAT pathway dysregulation and characterized by bone marrow fibrosis, cytopenias, and systemic inflammation [121] (Table 1). The Phase II MANIFEST trial explored pelabresib as monotherapy and in combination with ruxolitinib, a JAK inhibitor, demonstrating significant improvements in spleen volume, symptom burden, and bone marrow fibrosis, alongside reductions in inflammatory cytokines and mutant allele fractions. By targeting both oncogenic signaling and inflammatory pathways, pelabresib offers a dual mechanism to address unmet needs in MF.

Using matching-adjusted indirect comparisons (MAIC), clinical benefits relative to JAK inhibitor monotherapies, including ruxolitinib, momelotinib, and fedratinib were evaluated [122]. Results show significantly greater spleen volume reduction (SVR35) and symptom improvement (TSS50) at 24 weeks with pelabresib-ruxolitinib, highlighting its potential as a superior therapy.

The Phase III MANIFEST-2 study evaluated the efficacy and safety of pelabresib, a bromodomain and extraterminal domain (BET) inhibitor, in combination with ruxolitinib, as a first-line therapy for JAK inhibitor-naïve patients with myelofibrosis (MF). The study randomized 430 patients 1:1 to pelabresib plus ruxolitinib or placebo plus ruxolitinib, with a primary endpoint of spleen volume reduction (≥35%) at week 24. Pelabresib-ruxolitinib demonstrated a significantly higher spleen response rate (65.9% vs. 35.2%, *p* < 0.001) compared to placebo-ruxolitinib, with trends of improved symptom scores and reduced pro-inflammatory cytokine levels.

The combination therapy also resulted in meaningful improvements in bone marrow morphology, including reduced fibrosis and megakaryocyte density, and increased erythroid progenitor cell proportions. Hemoglobin response and reduced transfusion dependency further underscored its potential clinical benefits. Adverse events, such as thrombocytopenia and anemia, were manageable with dose modifications.

These findings establish pelabresib-ruxolitinib as a novel therapeutic approach that surpasses the standard JAK inhibitor monotherapy, addressing key unmet needs in MF. By mitigating disease-associated inflammation and improving hematological and morphological outcomes, this combination offers a significant advancement in the treatment landscape for MF patients. Ongoing evaluations will determine its long-term efficacy and survival benefits [123].

***BMS-986158***, a potent BET inhibitor, demonstrated promising efficacy in preclinical and early clinical trials for myelofibrosis (MF) when combined with JAK inhibitors (ruxolitinib or fedratinib) (Table 1). The Phase I/II CA011-023 study showed substantial spleen volume reductions (SVR35) in 73% of treatment-naïve and 58% of relapsed/refractory MF patients at 12 weeks. Disease modification was indicated by reductions in JAK2V617F allele burden. However, treatment-related adverse events, including thrombocytopenia, neutropenia, and anemia, required management. Despite encouraging results, development for myeloproliferative neoplasms (MPNs) was recently discontinued, highlighting challenges in advancing BET inhibitors for clinical use [124, 125].

**Histone deacetylase inhibitors (HDACi)** are widely studied agents in myeloproliferative neoplasms (MPNs), targeting both epigenetic dysregulation and inflammatory pathways. Preclinical mechanisms include the modulation of JAK/STAT signaling, suppression of inflammatory cytokines, and reversal of fibrosis [126]. Synergistic effects with JAK inhibitors, such as ruxolitinib, show promise in myelofibrosis, addressing key unmet needs like bone marrow remodeling and disease progression.

In MPN, a recent pre-clinical study identified HDAC11 as a critical driver of oncogenic hematopoiesis in while demonstrating its dispensability for normal hematopoiesis [127]. Using selective HDAC inhibitors and HDAC11-deficient mouse models, the researchers reveal that HDAC11 modulates megakaryocyte expansion, splenic architecture, and fibrosis in JAK2/MPL-driven MPNs. HDAC11 inhibition impairs the survival and proliferation of MPN cells without affecting normal hematopoietic cells, highlighting its therapeutic potential. The findings establish HDAC11 as a novel, selective target in MPNs, offering a promising avenue to overcome the limitations of current JAK inhibitors and address malignant hematopoiesis and fibrosis effectively. Likewise, HDAC8 has been identified as a therapeutic target in JAK2V617F-positive MPN, in both, hematopoietic and stromal compartments. HDAC8 is overexpressed in mesenchymal stromal cells (MSC) from MPN patients, supporting neoplastic hematopoiesis. Selective HDAC8 inhibition using PCI34051 impairs MSC-driven hematopoietic support, induces apoptosis in MSC and malignant hematopoietic cells, and reduces STAT3/STAT5 signaling [128].

Preclinical studies in myeloproliferative neoplasms (MPNs) have evaluated several histone deacetylase inhibitors (HDACs), including panobinostat, vorinostat, givinostat, and PCI34051 (HDAC8-specific) (Table 1). These inhibitors target class I and II HDACs, modulating epigenetic dysregulation and suppressing oncogenic JAK/STAT signaling. Mechanistically, they reduce pro-inflammatory cytokine production, promote apoptosis, and mitigate bone marrow fibrosis. Notably, panobinostat and givinostat demonstrate synergistic effects with JAK inhibitors, while HDAC8 inhibition selectively disrupts malignant stromal and hematopoietic interactions. These findings establish HDAC inhibitors as promising candidates to address MPN-associated inflammation, fibrosis, and disease progression.

***Panobinostat****,* a pan-HDAC inhibitor, and TG101209, a JAK2 inhibitor, have been studied in targeting JAK2V617F-positive myeloproliferative neoplasms (MPNs). Panobinostat disrupts JAK2-hsp90 interactions, leading to proteasomal degradation of JAK2, while TG101209 inhibits JAK2 autophosphorylation [129]. Combined treatment enhances apoptosis, significantly reduces JAK/STAT signaling, and demonstrates selective cytotoxicity against mutant JAK2-expressing cells compared to normal progenitors. These findings highlight the therapeutic potential of combining HDAC and JAK inhibitors in addressing disease progression and resistance in MPNs, providing a strong rationale for future clinical investigations.

In a Phase I trial, panobinostat demonstrated tolerability and clinical activity, with dose-limiting thrombocytopenia at 30 mg thrice weekly identified as a challenge, but lower doses (25 mg thrice weekly) were well tolerated. Among 18 patients, three achieved clinical improvement with significant reductions in palpable splenomegaly and anemia improvement. One patient achieved near-complete remission after 15 cycles, and marrow fibrosis resolution occurred after 16 cycles [130–132]. A Phase II study evaluated 40 mg thrice weekly panobinostat in MF patients, showing inhibition of JAK/STAT signaling, reduced JAK2V617F allele burden, and decreased inflammatory cytokines. However, limited clinical efficacy was observed due to poor tolerability, with only 16 of 35 patients completing two treatment cycles. A single patient met the International Working Group (IWG-MRT) criteria for response. Combination therapy trials with ruxolitinib showed synergistic efficacy in spleen volume reduction (SVR35 in 39%) and prolonged treatment durations up to five years. However, anemia, diarrhea, and thrombocytopenia were common toxicities. This combination achieved a maximum tolerated dose (MTD) of panobinostat 25 mg thrice weekly and ruxolitinib 15 mg twice daily [133].

***Givinostat*** is a pan-HDAC inhibitor with activity against class I and II histone deacetylases, designed to modulate epigenetic dysregulation and inflammatory signaling in myeloproliferative neoplasms (MPNs). By suppressing aberrant cytokine production, reducing JAK-STAT pathway hyperactivation, and mitigating fibrosis, it aims to address key pathological features of MPNs and improve clinical outcomes.

Recent studies investigated the effects of Givinostat (ITF2357) on JAK2V617F-positive myeloproliferative neoplasms (MPNs). Givinostat selectively inhibited proliferation, induced apoptosis, and reduced erythroid differentiation in JAK2-mutated cells by targeting key hematopoietic transcription factors, NFE2 and C-MYB [134]. These effects occur through both JAK2/STAT5 pathway inhibition and epigenetic modulation of histone acetylation. Givinostat demonstrates preferential activity against mutant over wild-type JAK2 cells and effectively suppresses inflammatory cytokine production. These findings highlight its dual mechanism of action and therapeutic potential for overcoming disease progression and inflammation in MPNs, supporting its ongoing clinical evaluation. Givinostat has demonstrated significant efficacy and tolerability in the treatment of myeloproliferative neoplasms (MPNs), particularly polycythemia vera (PV). Its dual action, targeting both inflammatory pathways and the JAK2/STAT5 signaling cascade, underpins its therapeutic potential [135]. In a Phase II trial (NCT00928707) evaluating givinostat in combination with hydroxycarbamide (HC) for HC-refractory PV patients, overall response rates (ORRs) were 55% and 50% at doses of 50 and 100 mg/day, respectively, with marked improvements in pruritus resolution (64–67%), hematologic control, and spleen size reduction. Grade 3 adverse events (AEs) were infrequent (4.5%), and treatment discontinuation occurred in only 18% of patients, highlighting its favorable safety profile. In subsequent studies, givinostat monotherapy and combination regimens demonstrated durable responses, with hematocrit normalization achieved in up to 56% of patients without phlebotomy dependence, along with significant reductions in platelet counts, white blood cell levels, and JAK2V617F allele burden [136]. The Phase Ib/II study (NCT01901432) established the maximum tolerated dose (100 mg twice daily), with 72.7–80.6% achieving complete or partial responses after 12–24 weeks. Furthermore, a long-term extension study (NCT01761968) confirmed sustained responses over a median of four years, with normalization of spleen size and elimination of pruritus in 89% of patients. A pivotal Phase III trial was planned to compare givinostat with hydroxycarbamide as a frontline treatment for high-risk PV patients (NCT04262141). This study aimed to evaluate givinostat’s efficacy in reducing thromboembolic events and delaying progression to myelofibrosis or AML. Ongoing investigations also include its application in essential thrombocythemia (ET) and combination regimens with other targeted therapies.

***Vorinostat***, a pan-HDAC inhibitor targeting class I and II histone deacetylases, is being developed for myeloproliferative neoplasms to address epigenetic dysregulation and inflammatory signaling. By promoting acetylation of histones and non-histone proteins, vorinostat modulates gene expression, induces apoptosis, and suppresses pro-inflammatory cytokine production, offering potential synergy with JAK inhibitors to mitigate fibrosis and disease progression in MPNs. Vorinostat has shown efficacy in preclinical models of myeloproliferative neoplasms, including essential thrombocythemia (ET) and polycythemia vera (PV). It reduces JAK-STAT pathway activation, induces apoptosis, and modulates gene expression, including upregulation of CDKN1A [137]. In vitro and in vivo, vorinostat decreases reactive oxygen species (ROS) and tumor burden while promoting apoptosis, particularly in granulocytic and monocytic lineages. A Phase II trial demonstrated normalization of blood counts and tumor burden reduction, though severe toxicities limited tolerability. Combining vorinostat with ROS-reducing agents enhances apoptosis synergistically, suggesting potential for improved therapeutic strategies. Another study found synergistic effects of ruxolitinib, a JAK1/2 inhibitor, and vorinostat, a histone deacetylase (HDAC) inhibitor, in treating JAK2V617F-positive myeloproliferative neoplasms (MPNs). Preclinical findings demonstrated enhanced apoptosis, G1-phase cell cycle arrest, and reduced colony-forming capacity in JAK2-mutant HEL cells and primary bone marrow mononuclear cells (BMMNCs) from MPN patients. Combination therapy significantly diminished phosphorylated STAT3 and AKT signaling and downregulated anti-apoptotic genes like BCL2. In clinical settings, the dual therapy improved efficacy, with stronger suppression of large colony-forming unit populations compared to monotherapy [138].

**Lysine-specific demethylase 1 (LSD1)** is an epigenetic regulator that modulates chromatin structure and gene expression through demethylation of histone H3 lysines [6]. LSD1 is overexpressed in MPNs, contributing to aberrant hematopoiesis and disease progression [139]. Inhibitors targeting LSD1 selectively impair the proliferation of malignant clones by restoring tumor suppressive pathways and inducing apoptosis, as shown in preclinical and clinical studies. These inhibitors also reduce mutant allele burden, bone marrow fibrosis, and inflammatory cytokines, demonstrating potential for disease modification in MPNs. The therapeutic efficacy of LSD1 inhibitors, such as bomedemstat, positions them as promising candidates for treating high-risk MPNs [118].

LSD1 inhibitors, particularly ***bomedemstat*** (IMG-7289) (Table 1), represent a novel therapeutic approach for MPNs, with significant progress made in their clinical development for essential thrombocythemia (ET) and (pre-fibrotic) myelofibrosis (MF) [140]. Bomedemstat is an irreversible inhibitor of LSD1, an epigenetic modulator essential for regulating chromatin architecture and gene expression. Clinical studies have demonstrated its potential to selectively target the malignant clone in MPNs, thereby reducing disease burden while sparing normal hematopoiesis.

In ET, bomedemstat has shown efficacy in normalizing platelet counts, alleviating symptoms, and reducing inflammatory cytokine levels. A Phase II trial (NCT04254978) demonstrated that patients treated with bomedemstat experienced durable hematologic responses, with improvements in total symptom scores (TSS) and no significant treatment-related cytopenias, a common challenge with current therapies.

In MF, bomedemstat has been evaluated in a Phase I/II trial (NCT03136185) enrolling patients who were refractory to or intolerant of JAK inhibitors like ruxolitinib or fedratinib. The trial revealed reductions in spleen volume, bone marrow fibrosis, and pro-inflammatory cytokines, with 25% of patients achieving a ≥ 50% reduction in TSS at week 12 and 14% attaining a ≥35% reduction in spleen volume (SVR35). Importantly, bomedemstat was well-tolerated, with dysgeusia being the most frequently reported adverse event, and no significant dose-limiting toxicities observed. Additionally, this agent reduced the mutant allele burden in approximately one-third of patients, suggesting its potential disease-modifying effects [141].

Bomedemstat’s mechanism of action, which involves increasing tumor suppressor protein p53 levels and reducing anti-apoptotic factors like BCL-xL, underpins its therapeutic efficacy. Its effects are further amplified by its ability to reduce megakaryocyte hyperplasia and inflammatory cytokine levels, key drivers of fibrosis and disease progression in MF [140].

Ongoing trials are extending the evaluation of LSD1 inhibitors. A global multicenter Phase II trial is currently assessing bomedemstat in a larger cohort of ET patients, including those resistant or intolerant to standard therapies. Moreover, combination studies are exploring the potential synergy between LSD1 inhibitors and other agents, such as JAK inhibitors, in MF to enhance outcomes and minimize toxicities [141].

These clinical advances underscore the transformative potential of LSD1 inhibitors as a targeted therapeutic strategy for MPNs. By addressing unmet needs such as mutant allele burden reduction, disease symptom alleviation, and fibrosis reversal, LSD1 inhibitors could redefine the treatment landscape for high-risk ET and MF, particularly for patients with limited therapeutic options.

**Hypomethylating agents (HMAs)**, such as azacitidine and decitabine, are increasingly utilized in MPNs and myelodysplastic/myeloproliferative (MDS/MPN) overlap neoplasms, particularly in advanced disease stages. These agents target aberrant DNA methylation, a hallmark of clonal evolution in these conditions, to modulate gene expression and promote hematopoietic rebalancing. HMAs are most commonly employed in hyperproliferative states, accelerated-phase MPNs, and blast-phase MPNs (MPN-BP), where leukemic transformation limits the efficacy of conventional therapies. Preclinical studies suggest that HMAs enhance the efficacy of JAK inhibitors by modulating cytokine signaling and epigenetic dysregulation, addressing both the proliferative and inflammatory components of disease.

A Phase II study was conducted to assess the safety and efficacy of ruxolitinib in combination with azacitidine in patients with myelofibrosis. Encouraging response rates were observed, with significant spleen volume reductions and improvements in bone marrow fibrosis, while manageable hematologic toxicities were reported [142]. Also, the efficacy and safety of ruxolitinib in combination with azacytidine were investigated in a phase II trial involving patients with myelodysplastic neoplasia / myeloproliferative neoplasms (MDS/MPNs) [143]. A response rate of 57% was observed, with improved outcomes particularly in patients with MDS/MPN-unclassifiable, while the combination was well tolerated, with manageable hematologic toxicities.

In ***accelerated and blast-phase MPNs***, azacitidine monotherapy has shown hematologic and clinical responses in approximately 30–40% of patients, with median overall survival ranging from 6–12 months. Azacitidine monotherapy has been investigated in patients with accelerated-phase (AP) and blast-phase (BP) myeloproliferative neoplasms (MPNs) who are ineligible for intensive chemotherapy or allogeneic hematopoietic cell transplantation [144–147]. A French national multicenter cohort study included 149 patients over 60 years old, treated with azacitidine alone (n = 60) or in combination with other agents (n = 89). The median overall survival (OS) for the entire cohort was 0.67 years, with a three-year OS of 13%. Patients receiving azacitidine monotherapy had a median OS of 0.58 years, compared to 0.84 years in those receiving combination therapies. The overall response rate was 54% for azacitidine monotherapy and 64% for combination therapies. Notably, patients treated with azacitidine alone were more likely to have complex karyotypes (53% vs. 30%) and TP53 mutations (44% vs. 19%), both associated with poorer outcomes [148]. Combination regimens with JAK inhibitors, such as ruxolitinib or fedratinib, have been explored to improve outcomes. A phase 1/2 clinical trial was conducted to evaluate the combination of ruxolitinib and decitabine in patients with post-myeloproliferative neoplasm acute myeloid leukemia (post-MPN AML) [149]. Despite a modest response rate and a median survival of 6.9 months, the combination was found to be tolerable, and a subset of patients who underwent allogeneic hematopoietic cell transplantation exhibited prolonged survival. The combination of ruxolitinib and decitabine was evaluated in a phase 2 trial for patients with myeloproliferative neoplasms in accelerated and blast phases. A response rate of 44% was reported, with a median overall survival of 9.5 months, indicating a potentially viable treatment option for this high-risk patient population [150]. Currently, the FAMY trial is a Phase I/II study evaluating the combination of oral azacitidine (CC-486) and fedratinib in patients with accelerated-phase (AP) and blast-phase (BP) MPNs. The trial aims to assess the safety, tolerability, and preliminary efficacy of this combination therapy. Patients receive oral azacitidine once daily on days 1-14 of a 28-day cycle, alongside fedratinib administered daily [151]. Clinical trials combining azacitidine with ruxolitinib demonstrated synergistic effects, leading to spleen volume reductions, improved symptom control, and molecular responses in 50–60% of patients. These combinations also showed promise in MDS/MPN overlap syndromes, such as chronic myelomonocytic leukemia (CMML), with response rates of up to 40% in selected cohorts. The combination of HMAs with venetoclax (HMA-Ven) has emerged as a promising approach, yielding response rates of 44–53% and a median OS of 7–9 months. This synergy is attributed to enhanced apoptosis via BCL-2 inhibition alongside epigenetic reprogramming. Intensive chemotherapy regimens, such as cytarabine and daunorubicin (7 + 3) or FLAG-IDA, achieve higher initial responses, with complete remission (CR) rates of up to 30% [144–147]. However, these strategies are limited by significant toxicity and short-lived responses unless followed by allogeneic hematopoietic stem cell transplantation (HSCT). A multicenter retrospective study evaluated venetoclax with azacitidine or decitabine in 32 patients with MPN-BP. Complete remission (CR) or CR with incomplete count recovery (CRi) was achieved in 44% of patients, with higher rates observed in the absence of complex karyotype or RAS mutations. The median overall survival was 8 months, and 43% of responders successfully underwent allogeneic hematopoietic stem cell transplantation. Compared to historical controls receiving hypomethylating agents alone, venetoclax-based combination therapy demonstrated superior response rates (44% vs. 4%) and median survival (8 vs. 5.5 months), supporting its potential as a bridge to transplantation [152]. Despite these advances, challenges such as treatment-related cytopenias and relapse underscore the need for optimized regimens and patient stratification to maximize clinical benefit. A study investigated the combination of interferon-alpha2 (IFN), azacitidine (Aza), and ruxolitinib for treating accelerated-phase (AP) and blast-phase (BP) MPN [153]. Azacitidine enhanced interferon signaling, reducing leukemia stem cell signatures and colony-forming potential by 50–70%. Preclinical data suggest the triple therapy synergistically targets malignant clones (via IFN and Aza) while ruxolitinib mitigates inflammation, offering a promising strategy to improve outcomes in treatment-resistant MPN.

## References

[1] Greaves M. Leukaemia ‘firsts’ in cancer research and treatment. Nat Rev Cancer. 2016;16:163–72. 26911190 10.1038/nrc.2016.3

[2] Cancer Genome Atlas Research N, Ley TJ, Miller C, Ding L, Raphael BJ, Mungall AJ, et al. Genomic and epigenomic landscapes of adult de novo acute myeloid leukemia. N Engl J Med. 2013;368:2059–74. 23634996 10.1056/NEJMoa1301689 PMC3767041

[3] Papaemmanuil E, Gerstung M, Bullinger L, Gaidzik VI, Paschka P, Roberts ND, et al. Genomic Classification and Prognosis in Acute Myeloid Leukemia. N Engl J Med. 2016;374:2209–21. 27276561 10.1056/NEJMoa1516192 PMC4979995

[4] Jones PA, Taylor SM. Cellular differentiation, cytidine analogs and DNA methylation. Cell. 1980;20:85–93. 6156004 10.1016/0092-8674(80)90237-8

[5] Feinberg AP, Vogelstein B. Hypomethylation distinguishes genes of some human cancers from their normal counterparts. Nature. 1983;301:89–92. 6185846 10.1038/301089a0

[6] Allis CD, Jenuwein T. The molecular hallmarks of epigenetic control. Nat Rev Genet. 2016;17:487–500. 27346641 10.1038/nrg.2016.59

[7] Spencer DH, Russler-Germain DA, Ketkar S, Helton NM, Lamprecht TL, Fulton RS, et al. CpG island hypermethylation mediated by DNMT3A is a consequence of AML progression. Cell. 2017;168:801–16.e13. 28215704 10.1016/j.cell.2017.01.021 PMC5328582

[8] Ley TJ, Ding L, Walter MJ, McLellan MD, Lamprecht T, Larson DE, et al. DNMT3A mutations in acute myeloid leukemia. N Engl J Med. 2010;363:2424–33. 21067377 10.1056/NEJMoa1005143 PMC3201818

[9] Challen GA, Sun D, Jeong M, Luo M, Jelinek J, Berg JS, et al. Dnmt3a is essential for hematopoietic stem cell differentiation. Nat Genet. 2011;44:23–31. 22138693 10.1038/ng.1009 PMC3637952

[10] Lu R, Wang P, Parton T, Zhou Y, Chrysovergis K, Rockowitz S, et al. Epigenetic perturbations by Arg882-Mutated DNMT3A potentiate aberrant stem cell gene-expression program and acute leukemia development. Cancer Cell. 2016;30:92–107. 27344947 10.1016/j.ccell.2016.05.008 PMC4945461

[11] Gaidzik VI, Weber D, Paschka P, Kaumanns A, Krieger S, Corbacioglu A, et al. DNMT3A mutant transcript levels persist in remission and do not predict outcome in patients with acute myeloid leukemia. Leukemia. 2018;32:30–7. 28643785 10.1038/leu.2017.200

[12] He YF, Li BZ, Li Z, Liu P, Wang Y, Tang Q, et al. Tet-mediated formation of 5-carboxylcytosine and its excision by TDG in mammalian DNA. Science. 2011;333:1303–7. 21817016 10.1126/science.1210944 PMC3462231

[13] Ito S, Shen L, Dai Q, Wu SC, Collins LB, Swenberg JA, et al. Tet proteins can convert 5-methylcytosine to 5-formylcytosine and 5-carboxylcytosine. Science. 2011;333:1300–3. 21778364 10.1126/science.1210597 PMC3495246

[14] Delhommeau F, Dupont S, Della Valle V, James C, Trannoy S, Masse A, et al. Mutation in TET2 in myeloid cancers. N Engl J Med. 2009;360:2289–301. 19474426 10.1056/NEJMoa0810069

[15] Pan X, Chang Y, Ruan G, Zhou S, Jiang H, Jiang Q, et al. TET2 mutations contribute to adverse prognosis in acute myeloid leukemia (AML): results from a comprehensive analysis of 502 AML cases and the Beat AML public database. Clin Exp Med. 2024;24:35. 38349460 10.1007/s10238-024-01297-0 PMC10864580

[16] Patnaik MM, Zahid MF, Lasho TL, Finke C, Ketterling RL, Gangat N, et al. Number and type of TET2 mutations in chronic myelomonocytic leukemia and their clinical relevance. Blood Cancer J. 2016;6:e472. 27662201 10.1038/bcj.2016.82 PMC5056973

[17] Bejar R, Lord A, Stevenson K, Bar-Natan M, Perez-Ladaga A, Zaneveld J, et al. TET2 mutations predict response to hypomethylating agents in myelodysplastic syndrome patients. Blood. 2014;124:2705–12. 25224413 10.1182/blood-2014-06-582809 PMC4208285

[18] Belizaire R, Wong WJ, Robinette ML, Ebert BL. Clonal haematopoiesis and dysregulation of the immune system. Nat Rev Immunol. 2023;23:595–610. 36941354 10.1038/s41577-023-00843-3 PMC11140722

[19] Paschka P, Schlenk RF, Gaidzik VI, Habdank M, Kronke J, Bullinger L, et al. IDH1 and IDH2 mutations are frequent genetic alterations in acute myeloid leukemia and confer adverse prognosis in cytogenetically normal acute myeloid leukemia with NPM1 mutation without FLT3 internal tandem duplication. J Clin Oncol. 2010;28:3636–43. 20567020 10.1200/JCO.2010.28.3762

[20] Dang L, White DW, Gross S, Bennett BD, Bittinger MA, Driggers EM, et al. Cancer-associated IDH1 mutations produce 2-hydroxyglutarate. Nature. 2009;462:739–44. 19935646 10.1038/nature08617 PMC2818760

[21] Ward PS, Patel J, Wise DR, Abdel-Wahab O, Bennett BD, Coller HA, et al. The common feature of leukemia-associated IDH1 and IDH2 mutations is a neomorphic enzyme activity converting alpha-ketoglutarate to 2-hydroxyglutarate. Cancer Cell. 2010;17:225–34. 20171147 10.1016/j.ccr.2010.01.020 PMC2849316

[22] Figueroa ME, Abdel-Wahab O, Lu C, Ward PS, Patel J, Shih A, et al. Leukemic IDH1 and IDH2 mutations result in a hypermethylation phenotype, disrupt TET2 function, and impair hematopoietic differentiation. Cancer Cell. 2010;18:553–67. 21130701 10.1016/j.ccr.2010.11.015 PMC4105845

[23] DiNardo CD, Stein EM, de Botton S, Roboz GJ, Altman JK, Mims AS, et al. Durable Remissions with Ivosidenib in IDH1-Mutated Relapsed or Refractory AML. N Engl J Med. 2018;378:2386–98. 29860938 10.1056/NEJMoa1716984

[24] Stein EM, DiNardo CD, Pollyea DA, Fathi AT, Roboz GJ, Altman JK, et al. Enasidenib in mutant IDH2 relapsed or refractory acute myeloid leukemia. Blood. 2017;130:722–31. 28588020 10.1182/blood-2017-04-779405 PMC5572791

[25] Meyer C, Larghero P, Lopes BA, Marschalek R. The KMT2A/MLL consensus gene structure: a comprehensive update for research and diagnostic implications. Leukemia. 2024;38:1403–6. 38678092 10.1038/s41375-024-02261-3 PMC11147768

[26] Krivtsov AV, Armstrong SA. MLL translocations, histone modifications and leukaemia stem-cell development. Nat Rev Cancer. 2007;7:823–33. 17957188 10.1038/nrc2253

[27] Krivtsov AV, Hoshii T, Armstrong SA. Mixed-lineage leukemia fusions and chromatin in leukemia. Cold Spring Harb Perspect Med. 2017;7:a026658. 10.1101/cshperspect.a026658 PMC5666623 28242784

[28] Somervaille TC, Matheny CJ, Spencer GJ, Iwasaki M, Rinn JL, Witten DM, et al. Hierarchical maintenance of MLL myeloid leukemia stem cells employs a transcriptional program shared with embryonic rather than adult stem cells. Cell Stem Cell. 2009;4:129–40. 19200802 10.1016/j.stem.2008.11.015 PMC2670853

[29] Krivtsov AV, Twomey D, Feng Z, Stubbs MC, Wang Y, Faber J, et al. Transformation from committed progenitor to leukaemia stem cell initiated by MLL-AF9. Nature. 2006;442:818–22. 16862118 10.1038/nature04980

[30] Meyer C, Larghero P, Almeida Lopes B, Burmeister T, Groger D, Sutton R, et al. The KMT2A recombinome of acute leukemias in 2023. Leukemia. 2023;37:988–1005. 37019990 10.1038/s41375-023-01877-1 PMC10169636

[31] Luo Z, Lin C, Shilatifard A. The super elongation complex (SEC) family in transcriptional control. Nat Rev Mol Cell Biol. 2012;13:543–7. 22895430 10.1038/nrm3417

[32] Somervaille TC, Cleary ML. Grist for the MLL: how do MLL oncogenic fusion proteins generate leukemia stem cells?. Int J Hematol. 2010;91:735–41. 20454944 10.1007/s12185-010-0579-8

[33] Wong NM, So CWE. Novel therapeutic strategies for MLL-rearranged leukemias. Biochim Biophys Acta Gene Regul Mech. 2020;1863:194584. 32534041 10.1016/j.bbagrm.2020.194584

[34] Thakker RV. Multiple endocrine neoplasia type 1 (MEN1) and type 4 (MEN4). Mol Cell Endocrinol. 2014;386:2–15. 23933118 10.1016/j.mce.2013.08.002 PMC4082531

[35] Huang J, Gurung B, Wan B, Matkar S, Veniaminova NA, Wan K, et al. The same pocket in menin binds both MLL and JUND but has opposite effects on transcription. Nature. 2012;482:542–6. 22327296 10.1038/nature10806 PMC3983792

[36] Yokoyama A, Cleary ML. Menin critically links MLL proteins with LEDGF on cancer-associated target genes. Cancer Cell. 2008;14:36–46. 18598942 10.1016/j.ccr.2008.05.003 PMC2692591

[37] Yokoyama A, Somervaille TC, Smith KS, Rozenblatt-Rosen O, Meyerson M, Cleary ML. The menin tumor suppressor protein is an essential oncogenic cofactor for MLL-associated leukemogenesis. Cell. 2005;123:207–18. 16239140 10.1016/j.cell.2005.09.025

[38] Borkin D, He S, Miao H, Kempinska K, Pollock J, Chase J, et al. Pharmacologic inhibition of the Menin-MLL interaction blocks progression of MLL leukemia in vivo. Cancer Cell. 2015;27:589–602. 25817203 10.1016/j.ccell.2015.02.016 PMC4415852

[39] Kuhn MW, Song E, Feng Z, Sinha A, Chen CW, Deshpande AJ, et al. Targeting Chromatin Regulators Inhibits Leukemogenic Gene Expression in NPM1 Mutant Leukemia. Cancer Discov. 2016;6:1166–81. 27535106 10.1158/2159-8290.CD-16-0237 PMC5584808

[40] Klossowski S, Miao H, Kempinska K, Wu T, Purohit T, Kim E, et al. Menin inhibitor MI-3454 induces remission in MLL1-rearranged and NPM1-mutated models of leukemia. J Clin Invest. 2020;130:981–97. 31855575 10.1172/JCI129126 PMC6994154

[41] Krivtsov AV, Evans K, Gadrey JY, Eschle BK, Hatton C, Uckelmann HJ, et al. A Menin-MLL Inhibitor Induces Specific Chromatin Changes and Eradicates Disease in Models of MLL-Rearranged Leukemia. Cancer Cell. 2019;36:660–73.e11. 31821784 10.1016/j.ccell.2019.11.001 PMC7227117

[42] Kwon MC, Thuring JW, Querolle O, Dai X, Verhulst T, Pande V, et al. Preclinical efficacy of the potent, selective menin-KMT2A inhibitor JNJ-75276617 (bleximenib) in KMT2A- and NPM1-altered leukemias. Blood. 2024;144:1206–20. 38905635 10.1182/blood.2023022480 PMC11419783

[43] Uckelmann HJ, Kim SM, Wong EM, Hatton C, Giovinazzo H, Gadrey JY, et al. Therapeutic targeting of preleukemia cells in a mouse model of NPM1 mutant acute myeloid leukemia. Science. 2020;367:586–90. 32001657 10.1126/science.aax5863 PMC7754791

[44] Issa GC, Aldoss I, DiPersio J, Cuglievan B, Stone R, Arellano M, et al. The menin inhibitor revumenib in KMT2A-rearranged or NPM1-mutant leukaemia. Nature. 2023;615:920–4. 36922593 10.1038/s41586-023-05812-3 PMC10060155

[45] Issa GC, Aldoss I, Thirman MJ, DiPersio J, Arellano M, Blachly JS, et al. Menin Inhibition With Revumenib for KMT2A-Rearranged Relapsed or Refractory Acute Leukemia (AUGMENT-101). J Clin Oncol. 2025;43:75–84. 39121437 10.1200/JCO.24.00826 PMC11687943

[46] Neff T, Sinha AU, Kluk MJ, Zhu N, Khattab MH, Stein L, et al. Polycomb repressive complex 2 is required for MLL-AF9 leukemia. Proc Natl Acad Sci USA. 2012;109:5028–33. 22396593 10.1073/pnas.1202258109 PMC3324004

[47] Tanaka S, Miyagi S, Sashida G, Chiba T, Yuan J, Mochizuki-Kashio M, et al. Ezh2 augments leukemogenicity by reinforcing differentiation blockage in acute myeloid leukemia. Blood. 2012;120:1107–17. 22677129 10.1182/blood-2011-11-394932

[48] Mabe NW, Perry JA, Malone CF, Stegmaier K. Pharmacological targeting of the cancer epigenome. Nat Cancer. 2024;5:844–65. 38937652 10.1038/s43018-024-00777-2 PMC11936478

[49] Maiques-Diaz A, Somervaille TC. LSD1: biologic roles and therapeutic targeting. Epigenomics. 2016;8:1103–16. 27479862 10.2217/epi-2016-0009 PMC5066116

[50] Schenk T, Chen WC, Gollner S, Howell L, Jin L, Hebestreit K, et al. Inhibition of the LSD1 (KDM1A) demethylase reactivates the all-trans-retinoic acid differentiation pathway in acute myeloid leukemia. Nat Med. 2012;18:605–11. 22406747 10.1038/nm.2661 PMC3539284

[51] Harris WJ, Huang X, Lynch JT, Spencer GJ, Hitchin JR, Li Y, et al. The histone demethylase KDM1A sustains the oncogenic potential of MLL-AF9 leukemia stem cells. Cancer Cell. 2012;21:473–87. 22464800 10.1016/j.ccr.2012.03.014

[52] Jutzi JS, Kleppe M, Dias J, Staehle HF, Shank K, Teruya-Feldstein J, et al. LSD1 inhibition prolongs survival in mouse models of MPN by selectively targeting the disease clone. Hemasphere. 2018;2:e54. 31723778 10.1097/HS9.0000000000000054 PMC6745991

[53] Fiskus W, Sharma S, Shah B, Portier BP, Devaraj SGT, Liu K, et al. Highly effective combination of LSD1 (KDM1A) antagonist and pan-histone deacetylase inhibitor against human AML cells. Leukemia. 2017;31:1658. 28322226 10.1038/leu.2017.77

[54] Salamero O, Molero A, Perez-Simon JA, Arnan M, Coll R, Garcia-Avila S, et al. Iadademstat in combination with azacitidine in patients with newly diagnosed acute myeloid leukaemia (ALICE): an open-label, phase 2a dose-finding study. Lancet Haematol. 2024;11:e487–e98. 38824932 10.1016/S2352-3026(24)00132-7

[55] Wang X, Chen CW, Armstrong SA. The role of DOT1L in the maintenance of leukemia gene expression. Curr Opin Genet Dev. 2016;36:68–72. 27151433 10.1016/j.gde.2016.03.015

[56] Chen CW, Armstrong SA. Targeting DOT1L and HOX gene expression in MLL-rearranged leukemia and beyond. Exp Hematol. 2015;43:673–84. 26118503 10.1016/j.exphem.2015.05.012 PMC4540610

[57] Cutler JA, Perner F, Armstrong SA. Histone PTM crosstalk stimulates Dot1 Methyltransferase activity. Trends Biochem Sci. 2021;46:522–4. 33879367 10.1016/j.tibs.2021.04.001

[58] Stein EM, Garcia-Manero G, Rizzieri DA, Tibes R, Berdeja JG, Savona MR, et al. The DOT1L inhibitor pinometostat reduces H3K79 methylation and has modest clinical activity in adult acute leukemia. Blood. 2018;131:2661–9. 29724899 10.1182/blood-2017-12-818948 PMC6265654

[59] Migliori V, Muller J, Phalke S, Low D, Bezzi M, Mok WC, et al. Symmetric dimethylation of H3R2 is a newly identified histone mark that supports euchromatin maintenance. Nat Struct Mol Biol. 2012;19:136–44. 22231400 10.1038/nsmb.2209

[60] Antonysamy S, Bonday Z, Campbell RM, Doyle B, Druzina Z, Gheyi T, et al. Crystal structure of the human PRMT5:MEP50 complex. Proc Natl Acad Sci USA. 2012;109:17960–5. 23071334 10.1073/pnas.1209814109 PMC3497828

[61] Burgos ES, Wilczek C, Onikubo T, Bonanno JB, Jansong J, Reimer U, et al. Histone H2A and H4 N-terminal tails are positioned by the MEP50 WD repeat protein for efficient methylation by the PRMT5 arginine methyltransferase. J Biol Chem. 2015;290:9674–89. 25713080 10.1074/jbc.M115.636894 PMC4392268

[62] Pal S, Baiocchi RA, Byrd JC, Grever MR, Jacob ST, Sif S. Low levels of miR-92b/96 induce PRMT5 translation and H3R8/H4R3 methylation in mantle cell lymphoma. EMBO J. 2007;26:3558–69. 17627275 10.1038/sj.emboj.7601794 PMC1949000

[63] Pal S, Yun R, Datta A, Lacomis L, Erdjument-Bromage H, Kumar J, et al. mSin3A/histone deacetylase 2- and PRMT5-containing Brg1 complex is involved in transcriptional repression of the Myc target gene cad. Mol Cell Biol. 2003;23:7475–87. 14559996 10.1128/MCB.23.21.7475-7487.2003 PMC207647

[64] Kim H, Ronai ZA. PRMT5 function and targeting in cancer. Cell Stress. 2020;4:199–215. 32743345 10.15698/cst2020.08.228 PMC7380451

[65] Kryukov GV, Wilson FH, Ruth JR, Paulk J, Tsherniak A, Marlow SE, et al. MTAP deletion confers enhanced dependency on the PRMT5 arginine methyltransferase in cancer cells. Science. 2016;351:1214–8. 26912360 10.1126/science.aad5214 PMC4997612

[66] Lu X, Fernando TM, Lossos C, Yusufova N, Liu F, Fontan L, et al. PRMT5 interacts with the BCL6 oncoprotein and is required for germinal center formation and lymphoma cell survival. Blood. 2018;132:2026–39. 30082494 10.1182/blood-2018-02-831438 PMC6236466

[67] Tarighat SS, Santhanam R, Frankhouser D, Radomska HS, Lai H, Anghelina M, et al. The dual epigenetic role of PRMT5 in acute myeloid leukemia: gene activation and repression via histone arginine methylation. Leukemia. 2016;30:789–99. 26536822 10.1038/leu.2015.308 PMC8034866

[68] Radzisheuskaya A, Shliaha PV, Grinev V, Lorenzini E, Kovalchuk S, Shlyueva D, et al. PRMT5 methylome profiling uncovers a direct link to splicing regulation in acute myeloid leukemia. Nat Struct Mol Biol. 2019;26:999–1012. 31611688 10.1038/s41594-019-0313-z PMC6858565

[69] Liu F, Zhao X, Perna F, Wang L, Koppikar P, Abdel-Wahab O, et al. JAK2V617F-mediated phosphorylation of PRMT5 downregulates its methyltransferase activity and promotes myeloproliferation. Cancer Cell. 2011;19:283–94. 21316606 10.1016/j.ccr.2010.12.020 PMC4687747

[70] Jin Y, Zhou J, Xu F, Jin B, Cui L, Wang Y, et al. Targeting methyltransferase PRMT5 eliminates leukemia stem cells in chronic myelogenous leukemia. J Clin Invest. 2016;126:3961–80. 27643437 10.1172/JCI85239 PMC5096815

[71] Man CH, Lam W, Dang CC, Zeng XY, Zheng LC, Chan NN, et al. Inhibition of PLK4 remodels histone methylation and activates the immune response via the cGAS-STING pathway in TP53-mutated AML. Blood. 2023;142:2002–15. 37738460 10.1182/blood.2023019782

[72] Gold S, Shilatifard A. Therapeutic targeting of BET bromodomain and other epigenetic acetylrecognition domain-containing factors. Curr Opin Genet Dev. 2024;86:102181. 38564841 10.1016/j.gde.2024.102181

[73] Orsolic I, Carrier A, Esteller M. Genetic and epigenetic defects of the RNA modification machinery in cancer. Trends Genet. 2023;39:74–88. 36379743 10.1016/j.tig.2022.10.004

[74] Wiener D, Schwartz S. The epitranscriptome beyond m(6)A. Nat Rev Genet. 2021;22:119–31. 33188361 10.1038/s41576-020-00295-8

[75] Rossello-Tortella M, Ferrer G, Esteller M. Epitranscriptomics in Hematopoiesis and Hematologic Malignancies. Blood Cancer Discov. 2020;1:26–31. 34661141 10.1158/2643-3249.BCD-20-0032 PMC8447282

[76] Feng M, Xie X, Han G, Zhang T, Li Y, Li Y, et al. YBX1 is required for maintaining myeloid leukemia cell survival by regulating BCL2 stability in an m6A-dependent manner. Blood. 2021;138:71–85. 10.1182/blood.2020009676 PMC8667054 33763698

[77] Perner F, Schnoeder TM, Xiong Y, Jayavelu AK, Mashamba N, Santamaria NT, et al. YBX1 mediates translation of oncogenic transcripts to control cell competition in AML. Leukemia. 2022;36:426–37. 34465866 10.1038/s41375-021-01393-0 PMC8807392

[78] Jayavelu AK, Schnoder TM, Perner F, Herzog C, Meiler A, Krishnamoorthy G, et al. Splicing factor YBX1 mediates persistence of JAK2-mutated neoplasms. Nature. 2020;588:157–63. 33239784 10.1038/s41586-020-2968-3

[79] Moshitch-Moshkovitz S, Sevilla-Sharon M, Ashwal-Fluss R, Glick-Saar E, Rechavi G, Dominissini D. mRNA m6A detection. Nat Rev Methods Primers. 2024;4. 10.1038/s43586-024-00365-9.

[80] Perner F, Pahl HL, Zeiser R, Heidel FH. Malignant JAK-signaling: at the interface of inflammation and malignant transformation. Leukemia. 2025;39:1011–30. 10.1038/s41375-025-02569-8 PMC12055591 40140631

[81] Perner F, Perner C, Ernst T, Heidel FH. Roles of JAK2 in Aging, Inflammation, Hematopoiesis and Malignant Transformation. Cells. 2019;8:854. 10.3390/cells8080854 PMC6721738 31398915

[82] Dawson MA, Bannister AJ, Saunders L, Wahab OA, Liu F, Nimer SD, et al. Nuclear JAK2. Blood. 2011;118:6987–8. 22194397 10.1182/blood-2011-10-385278 PMC4729533

[83] He J, Zhang Y. Janus kinase 2: an epigenetic ‘writer’ that activates leukemogenic genes. J Mol Cell Biol. 2010;2:231–3. 20051435 10.1093/jmcb/mjp054

[84] Peeken JC, Jutzi JS, Wehrle J, Koellerer C, Staehle HF, Becker H, et al. Epigenetic regulation of NFE2 overexpression in myeloproliferative neoplasms. Blood. 2018;131:2065–73. 29519804 10.1182/blood-2017-10-810622 PMC5934799

[85] Griffiths DS, Li J, Dawson MA, Trotter MW, Cheng YH, Smith AM, et al. LIF-independent JAK signalling to chromatin in embryonic stem cells uncovered from an adult stem cell disease. Nat Cell Biol. 2011;13:13–21. 21151131 10.1038/ncb2135 PMC3008749

[86] Kim H, Kim D, Choi SA, Kim CR, Oh SK, Pyo KE, et al. KDM3A histone demethylase functions as an essential factor for activation of JAK2-STAT3 signaling pathway. Proc Natl Acad Sci USA. 2018;115:11766–71. 30377265 10.1073/pnas.1805662115 PMC6243239

[87] Staehle AM, Peeken JC, Vladimirov G, Hoeness ME, Bojtine Kovacs S, Karantzelis N, et al. The histone demethylase JMJD2C constitutes a novel NFE2 target gene that is required for the survival of JAK2(V617F) mutated cells. Leukemia. 2023;37:919–23. 36709354 10.1038/s41375-023-01826-y PMC10079541

[88] Ernst P, Schnoder TM, Huber N, Perner F, Jayavelu AK, Eifert T, et al. Histone demethylase KDM4C is a functional dependency in JAK2-mutated neoplasms. Leukemia. 2022;36:1843–9. 35654819 10.1038/s41375-022-01611-3 PMC9252905

[89] Paulson M, Pisharody S, Pan L, Guadagno S, Mui AL, Levy DE. Stat protein transactivation domains recruit p300/CBP through widely divergent sequences. J Biol Chem. 1999;274:25343–9. 10464260 10.1074/jbc.274.36.25343

[90] Bhattacharya S, Eckner R, Grossman S, Oldread E, Arany Z, D’Andrea A, et al. Cooperation of Stat2 and p300/CBP in signalling induced by interferon-alpha. Nature. 1996;383:344–7. 8848048 10.1038/383344a0

[91] Wojciak JM, Martinez-Yamout MA, Dyson HJ, Wright PE. Structural basis for recruitment of CBP/p300 coactivators by STAT1 and STAT2 transactivation domains. EMBO J. 2009;28:948–58. 19214187 10.1038/emboj.2009.30 PMC2670858

[92] Gnatovskiy L, Mita P, Levy DE. The human RVB complex is required for efficient transcription of type I interferon-stimulated genes. Mol Cell Biol. 2013;33:3817–25. 23878400 10.1128/MCB.01562-12 PMC3811876

[93] Kleppe M, Koche R, Zou L, van Galen P, Hill CE, Dong L, et al. Dual Targeting of oncogenic activation and inflammatory signaling increases therapeutic efficacy in myeloproliferative neoplasms. Cancer Cell. 2018;33:29–43.e7. 29249691 10.1016/j.ccell.2017.11.009 PMC5760343

[94] Wyspianska BS, Bannister AJ, Barbieri I, Nangalia J, Godfrey A, Calero-Nieto FJ, et al. BET protein inhibition shows efficacy against JAK2V617F-driven neoplasms. Leukemia. 2014;28:88–97. 23929215 10.1038/leu.2013.234

[95] Marie IJ, Chang HM, Levy DE. HDAC stimulates gene expression through BRD4 availability in response to IFN and in interferonopathies. J Exp Med. 2018;215:3194–212. 30463877 10.1084/jem.20180520 PMC6279398

[96] Wildschut MHE, Mena J, Dordelmann C, van Oostrum M, Hale BD, Settelmeier J, et al. Proteogenetic drug response profiling elucidates targetable vulnerabilities of myelofibrosis. Nat Commun. 2023;14:6414. 37828014 10.1038/s41467-023-42101-z PMC10570306

[97] Jeong JJ, Gu X, Nie J, Sundaravel S, Liu H, Kuo WL, et al. Cytokine-Regulated Phosphorylation and Activation of TET2 by JAK2 in Hematopoiesis. Cancer Discov. 2019;9:778–95. 30944118 10.1158/2159-8290.CD-18-1138 PMC6697164

[98] Kantarjian HM, Thomas XG, Dmoszynska A, Wierzbowska A, Mazur G, Mayer J, et al. Multicenter, randomized, open-label, phase III trial of decitabine versus patient choice, with physician advice, of either supportive care or low-dose cytarabine for the treatment of older patients with newly diagnosed acute myeloid leukemia. J Clin Oncol. 2012;30:2670–7. 22689805 10.1200/JCO.2011.38.9429 PMC4874148

[99] Dombret H, Seymour JF, Butrym A, Wierzbowska A, Selleslag D, Jang JH, et al. International phase 3 study of azacitidine vs conventional care regimens in older patients with newly diagnosed AML with >30% blasts. Blood. 2015;126:291–9. 25987659 10.1182/blood-2015-01-621664 PMC4504945

[100] Lubbert M, Wijermans PW, Kicinski M, Chantepie S, Van der Velden W, Noppeney R, et al. 10-day decitabine versus 3 + 7 chemotherapy followed by allografting in older patients with acute myeloid leukaemia: an open-label, randomised, controlled, phase 3 trial. Lancet Haematol. 2023;10:e879–e89. 37914482 10.1016/S2352-3026(23)00273-9

[101] DiNardo CD, Jonas BA, Pullarkat V, Thirman MJ, Garcia JS, Wei AH, et al. Azacitidine and Venetoclax in previously untreated acute myeloid leukemia. N Engl J Med. 2020;383:617–29. 32786187 10.1056/NEJMoa2012971

[102] Montesinos P, Recher C, Vives S, Zarzycka E, Wang J, Bertani G, et al. Ivosidenib and Azacitidine in IDH1-Mutated Acute Myeloid Leukemia. N Engl J Med. 2022;386:1519–31. 35443108 10.1056/NEJMoa2117344

[103] Garcia-Manero G, McCloskey J, Griffiths EA, Yee KWL, Zeidan AM, Al-Kali A, et al. Oral decitabine-cedazuridine versus intravenous decitabine for myelodysplastic syndromes and chronic myelomonocytic leukaemia (ASCERTAIN): a registrational, randomised, crossover, pharmacokinetics, phase 3 study. Lancet Haematol. 2024;11:e15–e26. 38135371 10.1016/S2352-3026(23)00338-1

[104] Geissler K, Koristek Z, Del Castillo TB, Novak J, Rodriguez-Macias G, Metzelder SK, et al. Oral decitabine/cedazuridine versus intravenous decitabine for acute myeloid leukaemia: A randomised, crossover, registration, pharmacokinetics study. Br J Haematol. 2024;205:1734–45. 39313917 10.1111/bjh.19741

[105] DiNardo CD, Schuh AC, Stein EM, Montesinos P, Wei AH, de Botton S, et al. Enasidenib plus azacitidine versus azacitidine alone in patients with newly diagnosed, mutant-IDH2 acute myeloid leukaemia (AG221-AML-005): a single-arm, phase 1b and randomised, phase 2 trial. Lancet Oncol. 2021;22:1597–608. 34672961 10.1016/S1470-2045(21)00494-0

[106] Stein EM, DiNardo CD, Fathi AT, Mims AS, Pratz KW, Savona MR, et al. Ivosidenib or enasidenib combined with intensive chemotherapy in patients with newly diagnosed AML: a phase 1 study. Blood. 2021;137:1792–803. 33024987 10.1182/blood.2020007233 PMC8020270

[107] Wang ES, Issa GC, Erba HP, Altman JK, Montesinos P, DeBotton S, et al. Ziftomenib in relapsed or refractory acute myeloid leukaemia (KOMET-001): a multicentre, open-label, multi-cohort, phase 1 trial. Lancet Oncol. 2024;25:1310–24. 39362248 10.1016/S1470-2045(24)00386-3

[108] Searle E, Recher C, Abdul-Hay M, Abedin S, Aldoss I, Alfonso Pierola A, et al. Bleximenib Dose Optimization and Determination of RP2D from a Phase 1 Study in Relapsed/Refractory Acute Leukemia Patients with KMT2A and NPM1 Alterations. Blood (66th ASH Annual Meeting Abstracts). 2024.

[109] Zeidner JF, Yuda J, Watts JM, Levis MJ, Erba HP, Fukushima K, et al. Phase 1 Results: First-in-Human Phase 1/2 Study of the Menin-MLL Inhibitor Enzomenib (DSP-5336) in Patients with Relapsed or Refractory Acute Leukemia. Blood (66th ASH Annual Meeting Abstracts). 2024.

[110] Recher C, O’Nions J, Aldoss I, Pierola AA, Allred A, Alonso-Dominguez JM, et al. Phase 1b Study of Menin-KMT2A Inhibitor Bleximenib in Combination with Intensive Chemotherapy in Newly Diagnosed Acute Myeloid Leukemia with KMT2Ar or NPM1 Alterations. Blood (66th ASH Annual Meeting Abstracts). 2024.

[111] Zeidner J, Lin TL, Welkie R, Madanat Y, Koenig K, Swords R, et al. Phase 1B Study of Azacitidine, Venetoclax and Revumenib in Newly Diagnosed Older Adults with Npm1 Mutated or KMT2A Rearranged Aml: Interim Results of dose Escalation From The Beataml Consortium. Hemasphere (EHA Annual Meeting Abstracts 2024). 2024.

[112] Watts J, Minden MD, Bachiashvili K, Brunner AM, Abedin S, Crossman T, et al. Phase I/II study of the clinical activity and safety of GSK3326595 in patients with myeloid neoplasms. Ther Adv Hematol. 2024;15:20406207241275376. 10.1177/20406207241275376 PMC11406655 39290981

[113] Haque T, Cadenas FL, Xicoy B, Alfonso-Pierola A, Platzbecker U, Avivi I, et al. Phase 1 study of JNJ-64619178, a protein arginine methyltransferase 5 inhibitor, in patients with lower-risk myelodysplastic syndromes. Leuk Res. 2023;134:107390. 37776843 10.1016/j.leukres.2023.107390

[114] Craddock CF, Houlton AE, Quek LS, Ferguson P, Gbandi E, Roberts C, et al. Outcome of Azacitidine Therapy in acute myeloid leukemia is not improved by concurrent vorinostat therapy but is predicted by a diagnostic molecular signature. Clin Cancer Res. 2017;23:6430–40. 28765326 10.1158/1078-0432.CCR-17-1423

[115] Garcia-Manero G, Abaza Y, Takahashi K, Medeiros BC, Arellano M, Khaled SK, et al. Pracinostat plus azacitidine in older patients with newly diagnosed acute myeloid leukemia: results of a phase 2 study. Blood Adv. 2019;3:508–18. 30760466 10.1182/bloodadvances.2018027409 PMC6391673

[116] Garcia-Manero G, Sekeres MA, Egyed M, Breccia M, Graux C, Cavenagh JD, et al. A phase 1b/2b multicenter study of oral panobinostat plus azacitidine in adults with MDS, CMML or AML with ⩽30% blasts. Leukemia. 2017;31:2799–806. 28546581 10.1038/leu.2017.159 PMC5729337

[117] Roboz GJ, Yee K, Verma A, Borthakur G, de la Fuente Burguera A, Sanz G, et al. Phase I trials of the lysine-specific demethylase 1 inhibitor, GSK2879552, as mono- and combination-therapy in relapsed/refractory acute myeloid leukemia or high-risk myelodysplastic syndromes. Leuk Lymphoma. 2022;63:463–7. 34927529 10.1080/10428194.2021.2012667

[118] Ling VY, Heidel FH, Bywater MJ. Pathogenesis and management of high molecular risk myeloproliferative neoplasms. Haematologica. 2025;110:863–76. 10.3324/haematol.2023.283987 PMC11959265 39633552

[119] Pemmaraju N, Verstovsek S, Mesa R, Gupta V, Garcia JS, Scandura JM, et al. Defining disease modification in myelofibrosis in the era of targeted therapy. Cancer. 2022;128:2420–32. 35499819 10.1002/cncr.34205 PMC9322520

[120] Kleppe M, Koche R, Zou L, van Galen P, Hill CE, Dong L, et al. Dual targeting of oncogenic activation and inflammatory signaling increases therapeutic efficacy in myeloproliferative neoplasms. Cancer Cell. 2018;33:785–7. 10.1016/j.ccell.2018.03.024 PMC5908465 29634952

[121] Ferreira Gomes G, Harrison C. Pelabresib (CPI-0610): An exciting novel drug for the treatment of myelofibrosis. Curr Hematol Malig Rep. 2023;18:113–20. 37195585 10.1007/s11899-023-00696-6

[122] Gupta V, Mascarenhas J, Kremyanskaya M, Rampal RK, Talpaz M, Kiladjian JJ, et al. Matching-adjusted indirect comparison of the pelabresib-ruxolitinib combination vs JAKi monotherapy in myelofibrosis. Blood Adv. 2023;7:5421–32. 37530627 10.1182/bloodadvances.2023010628 PMC10509667

[123] Rampal RK, Grosicki S, Chraniuk D, Abruzzese E, Bose P, Gerds AT, et al. Pelabresib plus ruxolitinib for JAK inhibitor-naive myelofibrosis: a randomized phase 3 trial. Nat Med. 2025. 10.1038/s41591-025-03572-3. Online ahead of print. 10.1038/s41591-025-03572-3 PMC12092244 40065169

[124] Hilton J, Cristea M, Postel-Vinay S, Baldini C, Voskoboynik M, Edenfield W, et al. BMS-986158, a small molecule inhibitor of the bromodomain and extraterminal domain proteins, in patients with selected advanced solid tumors: results from a Phase 1/2a Trial. Cancers. 2022;14:4079. 10.3390/cancers14174079 PMC9454848 36077617

[125] Lavie D, Ribrag V, Loschi M, Yannakou CD, Alwan M, Abulafia AS, et al. BMS-986158, a Potent BET Inhibitor, in combination with Ruxolitinib or Fedratinib in Patients (pts) with intermediate- or high-risk Myelo?brosis (MF): Updated Results from a Phase 1/2 Study. Blood (ASH Annu Meet Abstr). 2023;142:623–5.

[126] Bose P, Verstovsek S. Investigational histone deacetylase inhibitors (HDACi) in myeloproliferative neoplasms. Expert Opin Investig Drugs. 2016;25:1393–403. 10.1080/13543784.2016.1250882 PMC5384641 27756180

[127] Yue L, Sharma V, Horvat NP, Akuffo AA, Beatty MS, Murdun C, et al. HDAC11 deficiency disrupts oncogene-induced hematopoiesis in myeloproliferative neoplasms. Blood. 2020;135:191–207. 31750881 10.1182/blood.2019895326 PMC6966930

[128] Ramos TL, Sanchez-Abarca LI, Redondo A, Hernandez-Hernandez A, Almeida AM, Puig N, et al. HDAC8 overexpression in mesenchymal stromal cells from JAK2+ myeloproliferative neoplasms: a new therapeutic target?. Oncotarget. 2017;8:28187–202. 28390197 10.18632/oncotarget.15969 PMC5438642

[129] Wang Y, Fiskus W, Chong DG, Buckley KM, Natarajan K, Rao R, et al. Cotreatment with panobinostat and JAK2 inhibitor TG101209 attenuates JAK2V617F levels and signaling and exerts synergistic cytotoxic effects against human myeloproliferative neoplastic cells. Blood. 2009;114:5024–33. 19828702 10.1182/blood-2009-05-222133 PMC2788976

[130] DeAngelo DJ, Mesa RA, Fiskus W, Tefferi A, Paley C, Wadleigh M, et al. Phase II trial of panobinostat, an oral pan-deacetylase inhibitor in patients with primary myelofibrosis, post-essential thrombocythaemia, and post-polycythaemia vera myelofibrosis. Br J Haematol. 2013;162:326–35. 23701016 10.1111/bjh.12384

[131] DeAngelo DJ, Spencer A, Bhalla KN, Prince HM, Fischer T, Kindler T, et al. Phase Ia/II, two-arm, open-label, dose-escalation study of oral panobinostat administered via two dosing schedules in patients with advanced hematologic malignancies. Leukemia. 2013;27:1628–36. 23385375 10.1038/leu.2013.38

[132] Mascarenhas J, Lu M, Li T, Petersen B, Hochman T, Najfeld V, et al. A phase I study of panobinostat (LBH589) in patients with primary myelofibrosis (PMF) and post-polycythaemia vera/essential thrombocythaemia myelofibrosis (post-PV/ET MF). Br J Haematol. 2013;161:68–75. 23330839 10.1111/bjh.12220

[133] Harrison C, Heidel FH, Vannucchi AM, Kiladjian JJ, Hayat A, Passamonti F, et al. A Phase Ib Dose-finding Study of Panobinostat and Ruxolitinib in Myelofibrosis. Hemasphere. 2022;6:e757. 35935608 10.1097/HS9.0000000000000757 PMC9348858

[134] Amaru Calzada A, Todoerti K, Donadoni L, Pellicioli A, Tuana G, Gatta R, et al. The HDAC inhibitor Givinostat modulates the hematopoietic transcription factors NFE2 and C-MYB in JAK2(V617F) myeloproliferative neoplasm cells. Exp Hematol. 2012;40:634–45.e10. 22579713 10.1016/j.exphem.2012.04.007

[135] Chifotides HT, Bose P, Verstovsek S. Givinostat: an emerging treatment for polycythemia vera. Expert Opin Investig Drugs. 2020;29:525–36. 10.1080/13543784.2020.1761323 PMC7534842 32693648

[136] Finazzi G, Vannucchi AM, Martinelli V, Ruggeri M, Nobile F, Specchia G, et al. A phase II study of Givinostat in combination with hydroxycarbamide in patients with polycythaemia vera unresponsive to hydroxycarbamide monotherapy. Br J Haematol. 2013;161:688–94. 23573950 10.1111/bjh.12332

[137] Cardoso BA, Ramos TL, Belo H, Vilas-Boas F, Real C, Almeida AM. Vorinostat synergizes with antioxidant therapy to target myeloproliferative neoplasms. Exp Hematol. 2019;72:60–71.e11. 30769020 10.1016/j.exphem.2019.02.002

[138] Hao X, Xing W, Yuan J, Wang Y, Bai J, Bai J, et al. Cotargeting the JAK/STAT signaling pathway and histone deacetylase by ruxolitinib and vorinostat elicits synergistic effects against myeloproliferative neoplasms. Invest N. Drugs. 2020;38:610–20. 10.1007/s10637-019-00794-4 31227936

[139] Gill H. Lysine-Specific Demethylase 1 (LSD1/KDM1A) inhibition as a target for disease modification in myelofibrosis. cells. 2022;11:2107. 10.3390/cells11132107 PMC9265913 35805191

[140] Rienhoff HY Jr., Gill H. Bomedemstat as an investigative treatment for myeloproliferative neoplasms. Expert Opin Investig Drugs. 2023;32:879–86. 10.1080/13543784.2023.2267980 37804041

[141] Tremblay D, Mascarenhas J. Next generation therapeutics for the treatment of myelofibrosis. Cells. 2021;10:1034. 10.3390/cells10051034 PMC8146033 33925695

[142] Masarova L, Verstovsek S, Hidalgo-Lopez JE, Pemmaraju N, Bose P, Estrov Z, et al. A phase 2 study of ruxolitinib in combination with azacitidine in patients with myelofibrosis. Blood. 2018;132:1664–74. 30185431 10.1182/blood-2018-04-846626 PMC6265645

[143] Assi R, Kantarjian HM, Garcia-Manero G, Cortes JE, Pemmaraju N, Wang X, et al. A phase II trial of ruxolitinib in combination with azacytidine in myelodysplastic syndrome/myeloproliferative neoplasms. Am J Hematol. 2018;93:277–85. 29134664 10.1002/ajh.24972 PMC12376909

[144] Ajufo HO, Waksal JA, Mascarenhas JO, Rampal RK. Treating accelerated and blast phase myeloproliferative neoplasms: progress and challenges. Ther Adv Hematol. 2023;14:20406207231177282. 37564898 10.1177/20406207231177282 PMC10410182

[145] Davidson MB, Kennedy JA, Capo-Chichi JM, Shi Y, Xu W, Cheung V, et al. Outcomes of intensive and nonintensive blast-reduction strategies in accelerated and blast-phase MPN. Blood Adv. 2024;8:1281–94. 38170760 10.1182/bloodadvances.2023011735 PMC10918486

[146] Patel AA, Yoon JJ, Johnston H, Davidson MB, Shallis RM, Chen EC, et al. Treatment approach and outcomes of patients with accelerated/blast-phase myeloproliferative neoplasms in the current era. Blood Adv. 2024;8:3468–77. 38739724 10.1182/bloodadvances.2024012880 PMC11260843

[147] Tefferi A, Alkhateeb H, Gangat N. Blast phase myeloproliferative neoplasm: contemporary review and 2024 treatment algorithm. Blood Cancer J. 2023;13:108. 37460550 10.1038/s41408-023-00878-8 PMC10352315

[148] Orvain C, Tavitian S, Mediavilla C, Boyer F, Santagostino A, Venton G, et al. Outcome of patients with accelerated and blast-phase myeloproliferative neoplasms ineligible for intensive chemotherapy or allogeneic hematopoietic cell transplantation treated by azacitidine alone or in combination - a FIM study. Blood (ASH Annu Meet Abstr). 2024;144:3167. 10.1002/hem3.70202 PMC12410146 40918085

[149] Bose P, Verstovsek S, Cortes JE, Tse S, Gasior Y, Jain N, et al. A phase 1/2 study of ruxolitinib and decitabine in patients with post-myeloproliferative neoplasm acute myeloid leukemia. Leukemia. 2020;34:2489–92. 32099037 10.1038/s41375-020-0778-0 PMC12136008

[150] Mascarenhas JO, Rampal RK, Kosiorek HE, Bhave R, Hexner E, Wang ES, et al. Phase 2 study of ruxolitinib and decitabine in patients with myeloproliferative neoplasm in accelerated and blast phase. Blood Adv. 2020;4:5246–56. 33104796 10.1182/bloodadvances.2020002119 PMC7594401

[151] Al-Ali HK. Fedratinib in Combination with CC-486, a hypomethylating agent, in patients with accelerated phase myelofibrosis. 2021. Accession Number: 2021-003650-23. https://www.clinicaltrialsregister.eu/ctr-search/trial/2021-003650-23/DE.

[152] Gangat N, Guglielmelli P, Szuber N, Begna KH, Patnaik MM, Litzow MR, et al. Venetoclax with azacitidine or decitabine in blast-phase myeloproliferative neoplasm: A multicenter series of 32 consecutive cases. Am J Hematol. 2021;96:781–9. 33844862 10.1002/ajh.26186 PMC8251544

[153] Hasselbalch HC, Skov V, Kjaer L, Larsen MK. Proof of concept of triple COMBI therapy to prohibit MPN progression to AML. Br J Haematol. 2024;204:16–8. 37957927 10.1111/bjh.19173

